# A novel exosome based therapeutic intervention against neuroendocrine prostate cancer

**DOI:** 10.1038/s41598-024-53269-9

**Published:** 2024-02-02

**Authors:** Sharanjot Saini, Amritha Sreekumar, Sandip Nathani, Diana M. Asante, Matthew N. Simmons

**Affiliations:** 1 Department of Biochemistry and Molecular Biology, Augusta University, 1410 Laney Walker Boulevard, Augusta, GA 30912 USA; 2 Department of Urology, Augusta University, Augusta, GA USA

**Keywords:** Prostate cancer, Targeted therapies

## Abstract

Neuroendocrine prostate cancer (NEPC) is a highly lethal variant of castration-resistant prostate cancer (CRPC) with poor survival rates. Current treatment options for NEPC are limited to highly toxic platinum drugs highlighting the urgent need for new therapies. This study aimed to develop a novel therapeutic approach using engineered exosomes against NEPC. Exosomes were modified to target CEACAM5, an NEPC surface antigen, by attaching CEACAM5 antibodies to HEK293T exosomes. These exosomes were loaded with drugs inhibiting EZH2 and the androgen receptor (AR) as recent research shows a persistent role of AR in NEPC wherein it plays a concerted role with EZH2 in driving neuronal gene programs. In vitro experiments with NEPC cell lines demonstrated that CEACAM5-targeted exosomes were specifically taken up by NEPC cells, leading to reduced cellular viability and decreased expression of neuronal markers. Further in vivo tests using a NEPC patient-derived xenograft model (LuCaP145.1) showed significant tumor regression in mice treated with engineered exosomes compared to control mice receiving IgG-labeled exosomes. These results suggest that CEACAM5-engineered exosomes hold promise as a targeted therapy for NEPC. Importantly, our exosome engineering strategy is versatile and can be adapted to target various surface antigens in prostate cancer and other diseases.

## Introduction

Prostate cancer (PCa) is a leading cause of male-cancer related mortality in United States with an estimated 34,700 deaths predicted to occur in 2023 with most mortality resulting from advanced, metastatic disease^1^. Prostate cancer is an androgen-dependent disease, with androgens acting via binding to androgen receptor (AR)^2^. Therefore, ablation of androgens via androgen deprivation therapy (ADT) is the goal of first line treatment that results in cancer regression initially. However, 2–3 years post-ADT, the disease develops to castration-resistant prostate cancer (CRPC) that has limited therapeutic options and poor survival rates^2^. Next generation of AR pathway inhibitors such as enzalutamide (MDV3100/ENZ) and abiraterone (ABI) are used for men with metastatic and non-metastatic CRPC that improves survival initially^3,4^. However, CRPC patients develop drug resistance over a certain period owing to heterogeneous molecular mechanisms such as AR amplification, emergence of ligand-independent splice variants, AR bypass signaling or complete AR independence^5,6^. The latter involves trans-differentiation of CRPC-adenocarcinomas to neuroendocrine variant, referred to as neuroendocrine prostate cancer (NEPC) via a reversible process known as neuroendocrine differentiation (NED)^7,8^. As a result of NED, PCa cells show decreased expression of luminal lineage markers such as AR and prostate-specific antigen (PSA) coupled with increased expression of alternative neuroendocrine (NE) lineage markers such as enolase 2 (ENO2), chromogranin A (CHGA) and synaptophysin (SYP)^7,9^. NEPC is associated with the presence of visceral metastasis to liver, lung and central nervous system, apart from lytic bone metastases and low serum PSA levels relative to disease burden^7^. Owing to lack of AR signaling, these PCa variants are impervious to ADT and constitute an extremely aggressive variant of advanced CRPC with shorter survival times (< 1 year) and limited therapeutic options^7,8^. Prognosis of these variants is extremely poor owing to late recognition of the disease and heterogeneous clinical features^7,8^. Though NEPC is often recognized late in the disease course subsequent to treatment with ENZ and ABI, it can also arise in metastatic CRPC after primary docetaxel therapy or early on after ADT^7–9^. Furthermore, NEPC can also arise de novo as pure small cell carcinoma from rare neuroendocrine cell populations in the prostate though it is not clear if therapy-induced NED is the same disease as de novo small cell PCa^10^. NEPC patients are currently treated with platinum-based chemotherapy that is associated with toxicity and relapses^8^. Therefore, there is an urgent need to develop novel therapeutic interventions for this disease. Exosomes are nano-sized (typically between 30 and 150 nm in size) membranous extracellular vesicles (EVs)^11^ that carry a cargo of RNA, proteins and lipids and facilitate intercellular communication by transferring their cargo to recipient cells to modulate target cell functions^12,13^. Owing to the potential of exosomes to carry a repertoire of molecules, exosomes have been effectively exploited for therapy of various diseases^14,15^. Owing to their biocompatibility, low toxicity, low immunogenicity, high permeability and stability in biological fluids, exosome-based therapies have several advantages^15^. Furthermore, exosomes can be effectively engineered to carry drugs to specific sites for targeted drug delivery^15^.

It has been realized that emergence of therapy-induced NEPC involves a series of genetic and epigenetic alterations^16–20^. The key genetic events driving NEPC include loss of tumor suppressors retinoblastoma (*RB1*), tumor protein 53 (*TP53*) and phosphatase and tensin homolog (*PTEN*)^16–20^. Prostate cancer-specific *TMPRSS2-ERG* gene rearrangements^19^ are present in 40–50% of NEPC. Amplifications of *NMYC* and Aurora Kinase A (AURKA)^16–18,20^ have been reported in NEPC. AURKA is a cell cycle kinase that stabilizes N-Myc, preventing its degradation^17,18,21^. Epigenetic alterations including alterations in DNA methylation, chromatin accessibility, histone marks and SWI/SNF complex have been associated with therapy-induced NEPC^18,20,22,23^. We showed that neural transcription factor (TF) BRN4 interplays with TF BRN2 in regulating SOX2 expression^24^ and driving NEPC^23^. Enhancer of zeste homolog 2 (EZH2), the catalytic subunit of Polycomb repressive complex 2 (PRC2), is overexpressed in NEPC^16,24,25^. It deposits repressive histone modification (H3K27m3) to inhibit lineage-specifying factors and maintain pluripotency^26^. In NEPC progression, EZH2 cooperates with oncogenic N-Myc, driving neuronal transcriptional programs^18^. EZH2 maintains bivalent chromatin states at N-Myc-bound neuronal gene promoters and suppresses SOX2 due to RB1 loss^24,27^. Additionally, EZH2 is linked to cAMP-response element binding protein (CREB) activation in PCa^28^. CREB/EZH2 axis represses Thrombospondin 1 (TSP1), promoting angiogenesis and NE induction in PCa xenograft^28^. EZH2 inhibitors are potential NEPC treatment agents, resensitizing tumors to AR-signaling inhibitors in CRPC^16,29^. EZH2 knockdown reduces neuronal-associated pathways in NEPC organoids^30^. Furthermore, NEPC is associated with varying levels of AR expression^31^. It has been reported that > 50% of treatment resistant PCa with NE features retain nuclear AR^10,31–33^.

Cancer cells often display specific surface markers that offer opportunities for targeted drug delivery. NEPC is known to express the cell surface marker Carcinoembryonic antigen (CEACAM5)^34^, while losing expression of other markers such as Prostate Specific Membrane Antigen (PSMA)^35^, making PSMA directed therapies ineffective. In light of this, we designed a therapeutic strategy specifically for NEPC wherein we engineered exosomes to target CEACAM5 by decorating HEK293T exosomes with anti-CEACAM5 antibody. These CEACAM5-directed exosomes were loaded with a combination of drugs tazemetostat and enzalutamide for specific NEPC therapeutic targeting. The rationale of using this drug combination is that a recent study showed that upon androgen deprivation, AR cistrome undergoes alterations with changes in chromatin architecture guiding AR transcriptional rerouting^36^. This study showed that AR cooperates with Enhancer of Zeste Homolog 2 (EZH2) to regulate lineage plasticity^36^, with AR and EZH2 co-occupying the reprogrammed AR cistrome that leads to transcriptional modulation of stem cell and neuronal gene networks^36^. Tazemetostat is a small molecule selective and S-adenosyl methionine competitive inhibitor of histone methyltransferase EZH2^37^ with anti-neoplastic activity. We reasoned that targeted delivery of tazemetostat + enzalutamide via exosomes may be an effective therapeutic modality in NEPC wherein the toxic effects of these drugs to non-target organs can be minimized. We tested these engineered exosomes in vitro and in vivo in NEPC models. Our data support that CEACAM5-targeted drug-loaded exosomes qualify as a novel targeted therapy in NEPC. Importantly, this exosome-based strategy is versatile and adaptable. It can be used to target the heterogeneity of NEPC/CRPC. Moreover, exosomes provide a natural delivery system that is highly scalable and can be easily translated for human applications due to their low immunogenicity and non-toxicity^15^. This versatile strategy can also be employed to target surface antigens in various other diseases.

## Results

### Engineering of CEACAM5 targeted therapeutic exosomes for NEPC

**Figure 1:**
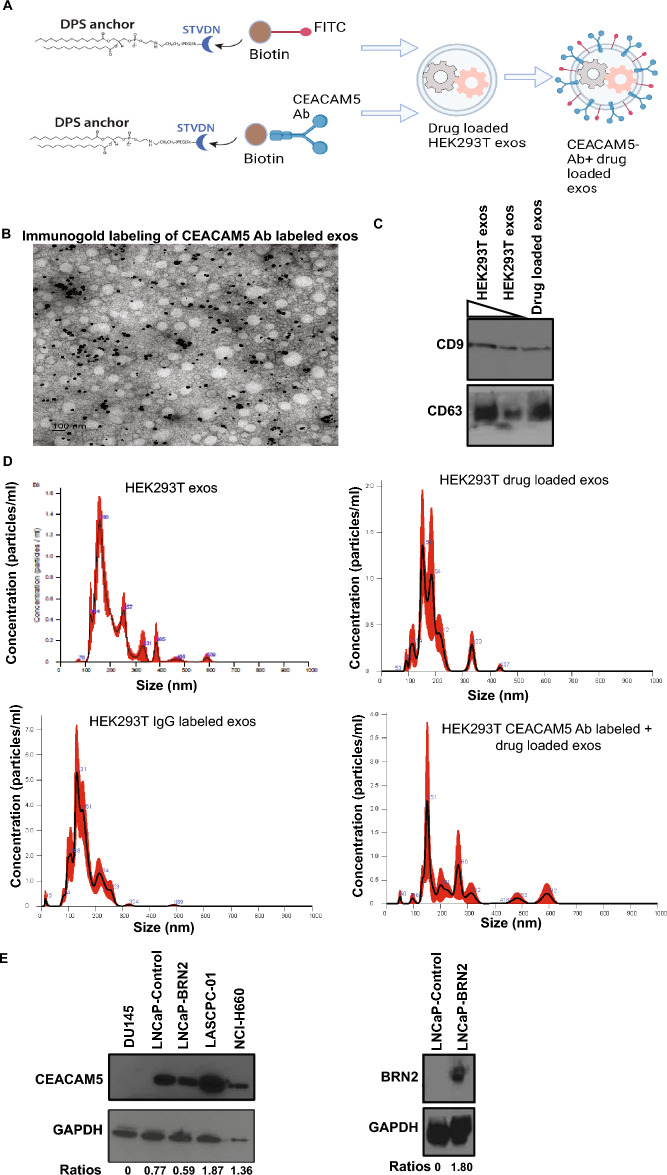
Engineering of therapeutic CEACAM5-targeted exosomes for NEPC. (A) Schematic of the design of CEACAM5 Ab decorated drug (tazemetostat + enzalutamide) loaded exosomes. In two separate reactions, DPS was conjugated with biotin-PEG-FITC (NANOCS, Inc.) and with biotin-CEACAM5 antibody/biotin-IgG. Reaction with biotin-CEACMA5 Ab is shown. Next, the complex was incubated with tazemetostat + enzalutamide labelled HEK 293T exosomes to generate CEACAM5 targeted drug loaded exosomes. (**B**) CEACAM5 Ab labeled exosomes were visualized by immunogold labeling with a rabbit secondary antibody followed by electron microscopy analyses. (**C**) Western blot analyses for exosomal markers CD9 and CD63 in two preparations of HEK293T exosomes (increasing concentrations) and drug loaded HEK293T exosomes. (**D**) Nanosight particle tracking analyses of HEK293T exosomes, HEK293T drug loaded exosomes, HEK293T IgG labelled exosomes, HEK293T CEACAM5 Ab + drug labelled exosomes showing the size of particles. Left panels: Western blot analyses for CEACAM5 expression in prostate cancer cell lines. (**E**) Western blot images were quantified by Image J. Values under the blot denotes normalized ratios of CEACAM5 expression. Statistical analyses of CEACAM5 expression from three biological replicates is represented in Fig. S1. Right panels: Western blot analyses of BRN2 expression in con/BRN2 overexpressing LNCaP cells.

With the aim of generating engineered CEACAM5 Ab labelled drug loaded exosomes for NEPC, we employed the strategy depicted in Fig. 1A. It has been previously reported that CEACAM5 is a specific NEPC surface marker^34^. To engineer CEACAM5 antibody-carrying exosomes, we employed a modular EV membrane anchoring platform consisting of streptavidin (STVDN) conjugated with 1,2-bis(dimethylphosphino)ethane: polyethylene glycol 5k (DMPE-PEG), referred to as DMPE-PEG-STVD. This platform enables attachment of biotin-labelled CEACAM5 antibody^38^ to the surface of tazemetostat + enzalutamide labelled HEK293T exosomes. In addition to antibody, biotin-FITC was incorporated on exosome surface to follow tracing of cellular uptake of exosomes in vitro and in vivo. As a control, IgG was incorporated on HEK293T exosomes along with biotin-FITC. CEACAM5 Ab labeled exosomes were visualized by immunogold labelling with a rabbit secondary antibody followed by electron microscopy analyses that confirmed the loading of CEACAM5 Ab on exosomes (Fig. 1B). To confirm the purity of exosomal preparations, we performed immunoblot analyses for exosomal markers CD9 and CD63 (Fig. 1C). Our analyses confirmed the presence of these markers in parental HEK293T exosomes and drug loaded exosomes. Engineered exosomes were also visualized and counted on Nanosight LM10 instrument (Fig. 1D). NTA analyses of HEK293T exosomes, including drug-loaded, IgG-loaded, and CEACAM5 antibody-loaded therapeutic exosomes, demonstrated the integrity of exosomes throughout the process of engineering them. We further checked CEACAM5 expression in NEPC cellular models by Western blot analyses (Fig. 1E, left panels). Our analyses confirmed a significant overexpression of CEACAM5 in NE cellular models NCI-H660 and LASCPC-01 while Du145 was CEACAM5 negative (Fig. 1E and Fig. S1). We also examined CEACAM5 expression in BRN2 overexpressing LNCaP cells as compared to LNCaP cells transfected with control construct. BRN2 overexpression was confirmed by Western blot analyses (Fig. 1E, right panel). LNCaP-BRN2 overexpressing cells had almost similar levels of CEACAM5 as LNCaP control (Fig. 1E, left panel) showing that mere overexpression of BRN2 may not be sufficient to drive NE phenotype.

### Combination of tazemetostat and enzalutamide delivered via exosomes reduce viability of neuroendocrine cellular models

**Figure 2:**
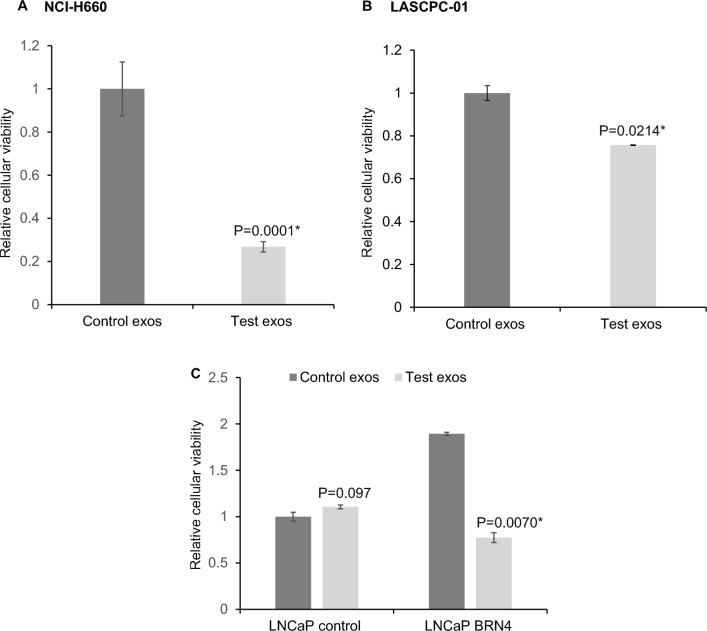
Combination of tazemetostat and enzalutamide delivered via exosomes are effective against NEPC. HEK293T exosomes were loaded with tazemetostat (1 µM) and enzalutamide (10 µM) by sonication. Purified control exosomes or drug loaded exosomes (10^8^ particles) were used for treating (**A**) NCI-H660, (**B**) LASCPC-01, (**C**) LNCaP cells expressing control/*BRN4* expressing construct for 4 days. Shown are the relative cellular viabilities in control exosome/drug loaded exosome treated cells as assessed by MTS cellular viability assay. The assay represented had three biological replicates and four technical replicates.

Given the combined roles of AR and EZH2 in driving neuronal gene programs in PCa^36^, we hypothesized that simultaneous inhibition of these molecules with a drug combination could be therapeutic in NEPC. Supporting this, a recent study found that combining the EZH2 inhibitor tazemetostat with the AR inhibitor enzalutamide had beneficial effects in NEPC^39^. To explore the therapeutic potential further, we investigated the effects of combined EZH2 and AR inhibition via exosomes in NEPC models (Fig. 2). HEK293T exosomes (exos) were loaded with tazemetostat (1 µM) and enzalutamide (10µM). Purified control exosomes or drug loaded exosomes (10^8^ particles) were used for treating NCI-H660 (Fig. 2A) and LASCPC-01 (Fig. 2B) cell lines for 4 days. As an additional NEPC model, we used *BRN4* expressing LNCaP cells (Fig. 2C)^23^ while LNCaP cells with a control construct were also included (Fig. 2C). Following exosome treatment, cellular viabilities were assessed by MTS cell viability assay. As compared to corresponding controls, treatment with drug loaded exosomes led to decreased cellular viabilities demonstrating that combination of tazemetostat and enzalutamide delivered to NEPC cells via exosomes possess therapeutic potential against NEPC. To demonstrate that the observed effects of these drugs are specific to NEPC, we also treated normal immortalized RWPE-1 cell line with control/tazemetostat + enzalutamide loaded exosomes (Fig. S2) under similar conditions as NCI-H660 cells. This assay showed that viability of RWPE-1 cells was unaffected by drug loaded exosome treatment supporting NEPC-specific effect of these drugs.

### CEACAM5-engineered exosomes specifically bind to NEPC cells in vitro

**Figure 3:**
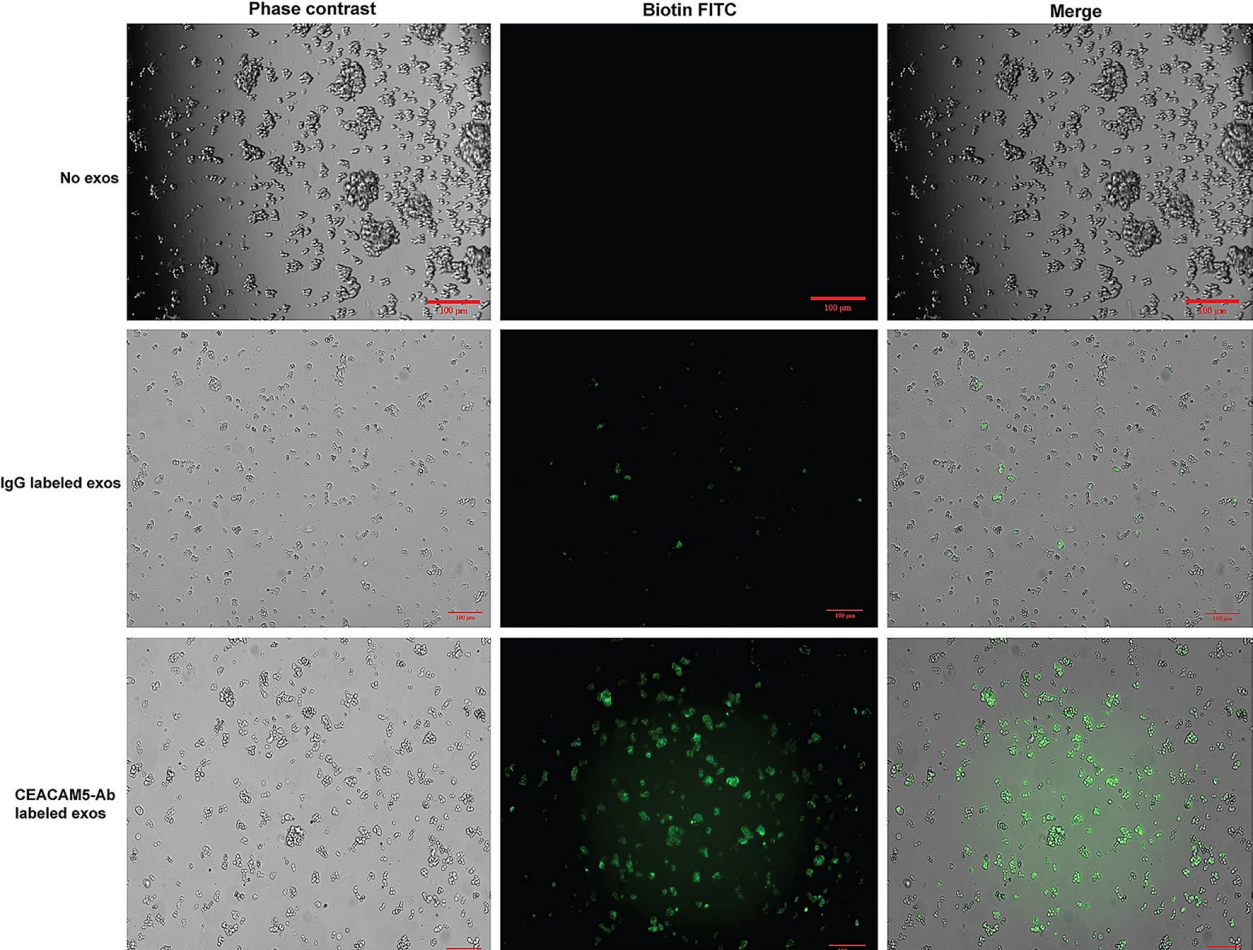
CEACAM5-engineered exosomes specifically bind to NEPC cells in vitro. NCI-H660 cells were treated with 10^8^ particles/ml of IgG (middle panels)/CEACAM5-Ab labelled HEK293T exosomes (lower panels) for 30 min. No exosome treated NCI-H660 cells were included as controls (upper panels). Following binding, cells were washed with PBS and visualized under Keyence microscope. Images were captured under × 10 magnification. Scale bar = 100 µm.

We next tested the specificity of CEACAM5 Ab engineered exosomes for NCI-H660 NE cells in vitro (Fig. 3) by employing an uptake assay. NCI-H660 cells were plated in 24-well culture dishes and treated with IgG + biotin-FITC-labelled/CEACAM5-Ab + biotin-FITC labelled HEK293T exosomes (10^8^ particles) for 30 min. As a control, no exosome treated NCI-H660 cells were included. Cells were harvested, washed with PBS, replated, and visualized under a fluorescent microscope. As shown in Fig. 3 (lower panels), NCI-H660 cells showed a specific uptake of CEACAM5-Ab + biotin-FITC labelled HEK293T exosomes compared to IgG and no exosome controls. To confirm that CEACAM5 targeted engineered exosomes are uptaken specifically to NEPC cells, we also performed binding assay with normal immortalized prostate epithelial cells RWPE-1 as described above for NCI-H660 cells (Fig. S3). This assay showed that NCI-H660 cells specifically uptake these engineered exosomes as no signal corresponding to exosome uptake was observed in RWPE-1 cells.

### Therapeutic CEACAM5-targeted exosomes reduce neuronal gene expression in NCI-H660 cells

**Figure 4:**
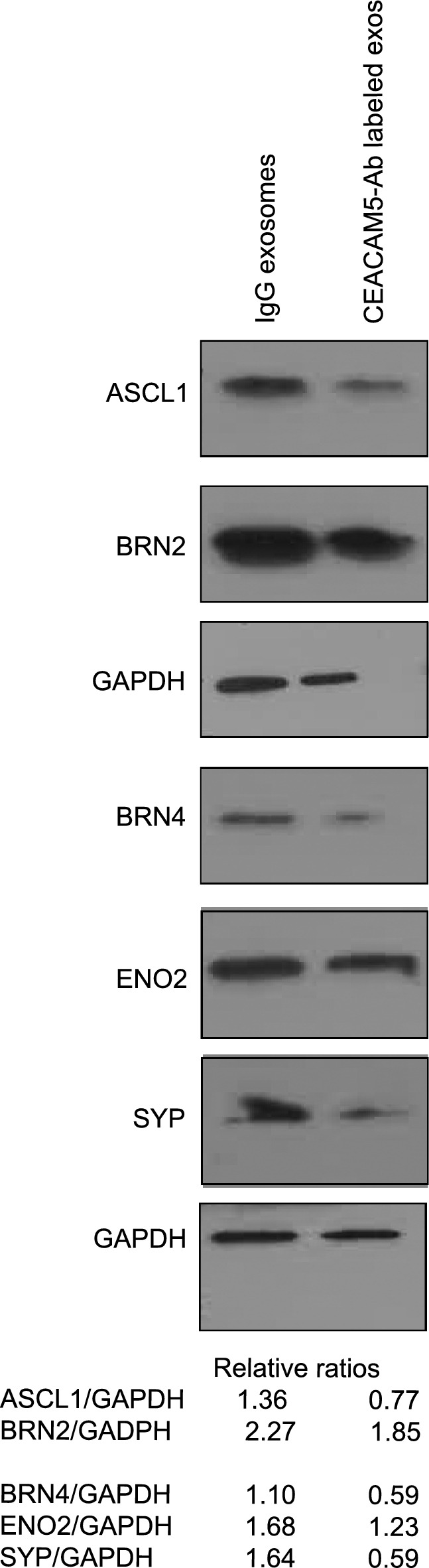
Therapeutic CEACAM5-targeted exosomes reduce neuronal gene expression in NCI-H660 cells. NCI-H660 cells were treated with 10^8^ particles/ml of IgG/CEACAM5 Ab tagged drug loaded exosomes for 4 days. Following the treatments, cell lysates were prepared and expression of neuronal markers ASCL1, BRN2, BRN4, ENO and SYP was assessed by Western blot analyses. GAPDH was used as a loading control. ‘exos’ refers to exosomes. All Western blot images were quantified by Image J Version 1.54d. Relative band intensities were calculated and are shown below the blots.

We next performed an in vitro experiment where we treated NCI-H660 cells with IgG control exosomes/CEACAM5 antibody targeted tazemetostat + enzalutamide loaded exosomes at a concentration of 10^8^ particles/ml media for 7 days. After treatment, cells were harvested and analyzed by Western blot analyses for neuronal markers and neuronal transcription factors (Fig. 4). Interestingly, we found a downregulation of neuronal markers ASCL1, BRN2, BRN4, ENO2 and SYP upon treatment with CEACAM5 targeted drug loaded exosomes as compared to control exosomes. This data lends support to our hypothesis that CEACAM5 targeted therapeutic exosomes inhibit neuroendocrine differentiation.

### Systemic administration of CEACAM5 targeting therapeutic exosomes induces tumor regression in LuCaP145.1 NEPC PDX model

**Figure 5:**
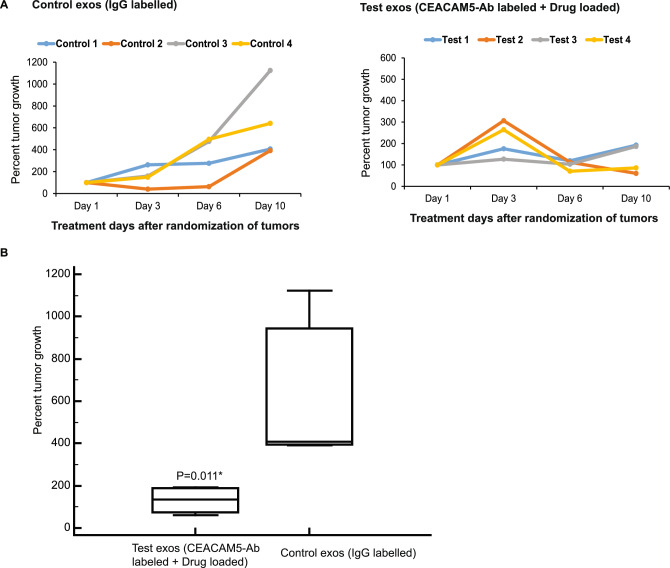
Therapeutic CEACAM5-targeted exosomes induce tumor regression in LuCaP145.1 NE PDX model. LuCaP145.1 PDX model was grown in male FOX-Chase SCID mice (n = 8). Once subcutaneous tumors were established, mice were randomized into two groups: Control and Test (n = 4/group). Test mice were administered 10^9 particles of engineered exosomes (CEACAM5 Ab labelled + tazemetostat + enzalutamide loaded HEK293T exosomes) while control mice were administered IgG labelled HEK293T exosomes via tail vein twice a week for 10 days. (**A**) Percent tumor growth for individual animals in Control group (left panel) and Test group (right panel) at indicated time points of exosome treatment. (**B**) Box plots showing average percent tumor growth in Test vs Control groups at Day 10 of exosome treatment. Statistical significance was calculated by One Way ANOVA (*P < 0.05).

To examine the effects of CEACAM5 Ab engineered exosomes in vivo, we employed LuCaP145.1 NE patient-derived xenograft (PDX) model. We established LuCaP145.1 tumors in FOX-Chase SCID mice. Once established, mice were divided into two groups: Control and Test (n = 4/group). Test mice were administered 10^9^ particles of engineered exosomes (CEACAM5 Ab labelled + tazemetostat + enzalutamide loaded HEK293T exosomes) via tail vein twice a week for 10 days. Controls included LuCaP145.1 xenografts treated with 10^9^ particles of control exosomes (IgG labelled HEK293T exosomes). Tumor volumes were measured regularly, and percent tumor growths were calculated (Fig. 5A,B). While the control mice showed increased tumor volumes with time, CEACAM5 Ab labelled, drug loaded exosome-treated mice showed significant regression of LuCaP145.1 tumors (P = 0.011*). To monitor potential off target effects, mice weight were monitored regularly (Fig. S4). The average mice weight was not altered significantly upon exosome treatment showing the absence of side effects of exosome treatment. To confirm that the observed therapeutic effects were due to specific uptake of exosomes by tumor cells, we performed bio-distribution studies (Fig. S5). Control IgG exosomes or CEACAM5 antibody loaded exosomes were radiolabeled with Iodine-131. Radiolabeled exosomes were administered via the tail vein into mice bearing LuCaP145.1 tumors followed by in vivo SPECT imaging after 3 h of exosome injections. We observed radioactive signals within LuCaP145.1 tumors in CEACAM5 antibody tagged mice showing the specific tumor uptake of these exosomes. These data support the therapeutic potential of CEACAM5 targeted engineered exosomes in NEPC.

### Intravenous injection of therapeutic CEACAM5-targeted exosomes reduces neuronal gene expression in LuCaP145.1 PDX model

**Figure 6:**
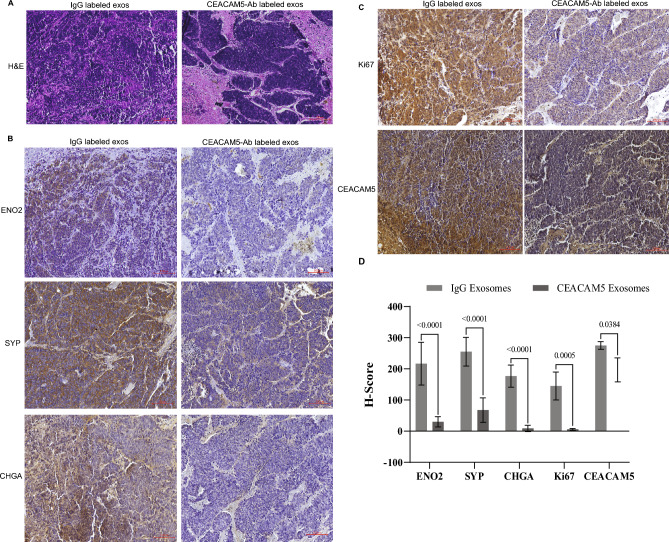
Intravenous injection of therapeutic CEACAM5-targeted exosomes reduce neuronal gene expression and reduce cellular proliferation in LuCaP145.1 PDX model. LuCaP145.1 xenografts treated with control exosomes (IgG labelled HEK293T exosomes) or test exosomes (CEACAM5 Ab labelled + tazemetostat + enzalutamide loaded HEK293T exosomes) systemically in in vivo* studies* (Fig. 5) were harvested. Tumors were embedded, sectioned, and stained. (**A**) H&E analysis of harvested tumors in control (left panel) and test (right panel) groups. (**B**) Immunohistochemical staining of ENO2, SYP and CHGA in control (left panel) and test (right panel) groups. (**C**) Immunohistochemical staining of CEACAM5 and Ki67 in control (left panel) and test (right panel) groups. (**D**) Quantification of staining intensities in control and test tumors.

We further harvested LuCaP145.1 PDX tumors from control/test exosome treated mice from in vivo studies described in Fig. 5. We examined histological changes induced by engineered exosomes by performing H&E staining (Fig. 6A). This staining showed that CEACAM5 Ab labelled + tazemetostat + enzalutamide loaded HEK293T exosome treated tumors showed altered morphology with overall decreased cellularity and areas of vacuolization as compared to control group. We performed immunohistochemical (IHC) staining for NE markers ENO2, SYP and CHGA (Fig. 6B,D). These analyses showed significantly decreased expression of these neuronal markers upon CEACAM5 Ab-engineered exosome treatment as compared to controls. The proliferative marker Ki67 was reduced upon treatment with CEACAM5 targeted drug loaded exosomes as compared to control IgG exosomes (Fig. 6C, upper panels and 6D). CEACAM5 staining (Fig. 6C, lower panels and 6D) showed that its expression was reduced in test exosome treated tumors as compared to control exosome treated tumors, supporting the suppression of neuroendocrine characteristics upon targeting exosome treatment. These data support the therapeutic potential of engineered exosomes in NEPC.

### Systemic administration of therapeutic CEACAM5-targeted exosomes induce apoptosis and enhance E-cadherin expression in LuCaP145.1 PDX model

**Figure 7:**
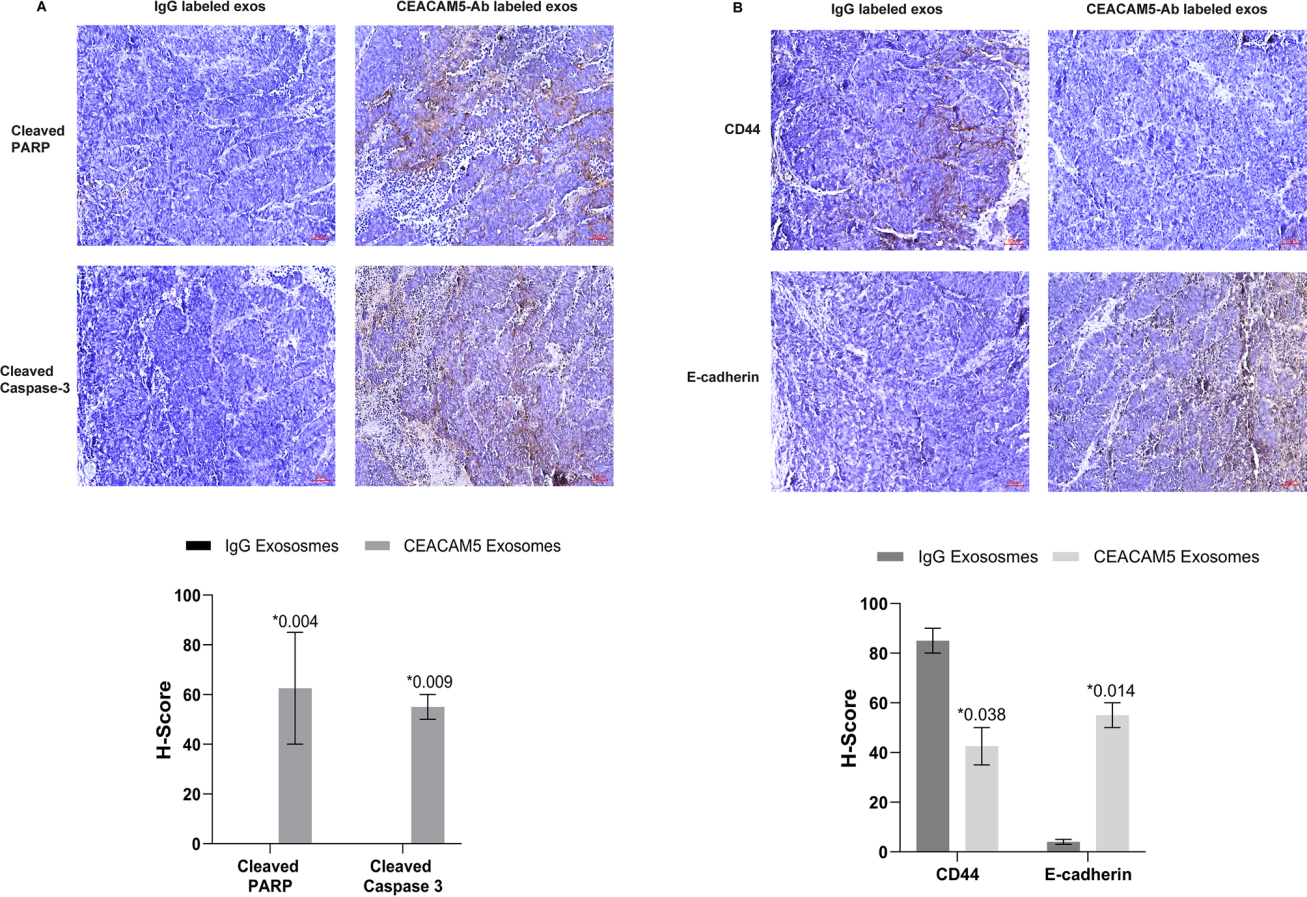
Systemic administration of therapeutic CEACAM5-targeted exosomes induce apoptosis and enhance E-cadherin expression in LuCaP145.1 PDX model. Control and test mice were administered 10^9^ particles of control exosomes (IgG labelled HEK293T exosomes) and engineered exosomes (CEACAM5 Ab labelled + tazemetostat + enzalutamide loaded HEK293T exosomes) respectively, via tail vein twice a week for 10 days. At the end of treatment, mice were sacrificed and tumors were harvested for immunohistochemical analysis. (**A**) Immunohistochemical staining of cleaved PARP and cleaved caspase-3 in control (left panel) and test (right panel) groups. (**B**) Immunohistochemical staining of CD44 and E-cadherin in control (left panel) and test (right panel) groups. Images were taken under × 20 magnification of inverted light microscope. Scale bar is 50 µm.

In view of the promising in vivo effects from systemic administration of therapeutic CEACAM5-targeted drug loaded exosomes, we sought to examine the mechanistic basis of these effects. Towards this, we further characterized the tumors from in vivo study. IHC analyses of apoptotic markers cleaved PARP and cleaved caspase 3 (Fig. 7A) showed that targeted exosomes induced significant apoptotic cell death in LuCaP145.1 xenografts as compared to control exosome treated xenografts. We also analyzed the expression of stem cell marker CD44 and epithelial marker E-cadherin in these tumors (Fig. 7B). We found that CD44 expression was observed in LuCaP145.1 xenografts treated with control exosomes (Fig. 7B, upper left panel). However, CEACAM5 targeted drug loaded exosome treated mice showed loss of CD44 expression (Fig. 7B, upper right panel). Contrary to this pattern, E-cadherin expression was observed to be increased in CEACAM5 targeted exosome treated xenografts as compared to control group (Fig. 7B, lower panels). These data support the therapeutic potential of CEACAM5-targeted exosomes.

## Discussion

We report to our knowledge for the first time, a novel exosome-based therapeutic strategy for neuroendocrine prostate cancer. Clinical management of neuroendocrine prostate cancer patients is currently challenging and survival rates for NEPC are dismal as there are limited therapeutic options for this disease^7,8^. These patients are currently treated with platinum-based chemotherapy that is associated with toxicity and relapses^12^. With the increased understanding of molecular underpinnings of neuroendocrine trans-differentiation, novel targeted therapies are being developed and several are in clinical trials^12^. AURKA inhibitor Alisertib that disrupts the interaction between N-Myc and AURKA has been examined as a therapeutic modality in NEPC^40^. However, this modality was found to be effective in a subset of patients. Since NEPC overexpresses DLL3, a cell surface ligand for the Notch receptor, a humanized DLL3 antibody has been exploited for therapeutic targeting of NEPC^25–27^. Targeting the epigenome via EZH2 inhibitors and BET (Bromodomain and Extraterminal family) inhibitors has shown promise in preclinical models of NEPC^18^. Though these therapeutic strategies are being developed, considering the heterogeneity of NEPC states, the targeted molecular alterations are often expressed in a subset of tumors, thereby rendering these therapies often effective in a small proportion of NEPC tumors. Moreover, several of the targeted therapies in development are associated with several adverse effects^35^. This emphasizes the urgent need for newer, more effective therapeutic interventions against this challenging disease.

Our data suggests that CEACAM5-targeted engineered exosomes loaded with EZH2 and AR inhibitors show promising therapeutic potential for NEPC. Decorating therapeutic exosomes with CEACAM5 antibody targets these exosomes to NEPC cells, as CEACAM5 is a specific cell surface marker for this PCa variant^34^. CEACAM5 is a glycophosphatidylinositol-anchored membrane protein that was recently identified as a potential immunotherapeutic target for NEPC^34^. It is a member of CEACAM family of cell adhesion molecules that regulate a variety of cellular processes such as proliferation, migration and metastasis^41^. CEACAM5 targeting chimeric antigen receptor T cell (CAR-T) cells^34^, antibody^42^ and antibody drug conjugate (ADC)^35^ have shown a potential benefit for NEPC. The advantages of our proposed exosome-based approach is ease of production, scalability and non-immunogenicity. Our versatile exosome engineering approach can target different surface antigens that can address the heterogeneity of NEPC and CRPC. We have successfully tested this strategy in our lab for metastatic CRPC using PSMA Ab along with EZH2 and AR inhibitors. It can be applied to target PSMA either alone or in combination with CEACAM5 by displaying biotinylated PSMA Ab and biotinylated CEACAM5 Ab on exosomes, offering flexibility in its application. Similar to our study, prior studies have demonstrated the therapeutic potential of engineered exosomes. Krishn et al. showed that αVβ6 integrin, an epithelial-specific cell surface receptor that is overexpressed in prostate cancer could be specifically targeted using small extracellular vesicles (sEVs)^43^. sEVs were engineered to carry ITGB6-specific siRNAs and were shown to inhibit adhesion and migration of recipient prostate cancer cells^43^.

Given the recent findings showing a persistent role of AR in NEPC wherein it plays a concerted role with EZH2 in driving neuronal gene programs in PCa^36^, the combined inhibition of AR and EZH2 by using a combination of EZH2 inhibitor tazemetostat with AR inhibitor enzalutamide has been found to be viable strategy in NEPC^39^. Tazemetostat is a small molecule selective and S-adenosyl methionine competitive inhibitor of histone methyltransferase EZH2^37^ with anti-neoplastic activity that is FDA approved for epithelioid sarcoma and follicular lymphoma. However, it is not yet approved for prostate cancer. EZH2 inhibitors have been shown to re-sensitize tumors to AR-signaling inhibitors in CRPC^16,29^ and EZH2 knockdown in NEPC organoids has been reported to repress neuronal associated pathways^30^. In this study, we found that a combination of these inhibitors delivered via exosomes exhibit therapeutic effects. We tested these engineered exosomes in vitro and in vivo in NEPC models. Importantly, our in vitro data shows the specific uptake of CEACAM5 engineered exosomes as compared to IgG-tagged exosomes. Further, systemic administration of CEACAM5 targeted exosomes induced tumor regression in LuCaP145.1 NE PDX model. Our in vitro and in vivo data support decreased expression of neuronal markers upon administration of targeted exosomes.

Our data suggests that our engineered exosomes induce anti-apoptotic and anti-proliferative effect. Enzalutamide and tazemetostat are known for their cytostatic effects. Our analyses show non-significant changes in apoptosis in PCa cell lines treated with this drug combination as compared to control exosome treated cells (not shown). However, when CEACAM5 antibody decorated drug loaded exosomes were administered systemically in vivo to mice bearing LuCaP145.1 xenografts, we saw increased apoptotic cells. The limitations of the study include use of a single NEPC patient-derived xenograft model for in vivo studies using immunodeficient mice. Future studies will expand the study to include other NEPC PDX models and immunologically competent genetically engineered mouse models. These studies would pave the way for clinical trials in humans. The strength of our approach is that exosomes can be engineered to carry a variety of cytotoxic/cytostatic drugs. Our adaptable and modular exosome-based platform can be easily customized to address the heterogeneity of NEPC states and to address various tumor-specific patient profiles. We propose that these targeting exosomes can be potentially loaded with a variety of drugs and/or molecules and can be directed to various surface antigens. Our strategy demonstrates that exosomes can be effectively engineered to carry drugs in a targeted manner that can potentially reduce the off-target effects of these drugs. We suggest that in view of varying AR independent CRPC variants observed recently^44^, these variants can be targeted by our platform by packaging specific drugs against these variants. This strategy can be translated to neuroendocrine tumors of other organs such as small cell neuroendocrine lung cancer that shares common susceptibilities to NEPC^45^. Further, this strategy that involves targeting antibodies against surface antigens on exosomes can be expanded for drug delivery for other diseases as well. Though we employed this strategy for therapeutic purposes, our strategy can also be modified for the development of a new diagnostic tool for NEPC. Considering the increasing incidences of therapy induced NEPC, the proposed project will have significant implications and will impact the clinical management of NEPC.

## Methods

### Cell lines and cell culture

Human embryonic kidney 293T (HEK293T) cells and prostate cancer cell lines Du145, LNCaP, NCI-H660 (CRl-5813) (25) and LASCPC-01 (CRL-3356) were procured from American Tissue Culture Collection Center (ATCC) and maintained under recommended conditions. All cell lines were maintained in an incubator with a humidified atmosphere of 95% air and 5% CO_2_ at 37 °C. The experiments with cell lines were performed within 6 months of their procurement/resuscitation. Prostate cell lines were authenticated by DNA short-tandem repeat analysis. pLenti-BRN4-GIII-CMV (#LV268693) and pLenti-BRN2-GIII-CMV (#LV268681) were procured from Applied Biological Materials and used for overexpressing BRN4 and BRN2, respectively in LNCaP-AR cell line followed by selection in 1 µg/ml puromycin in RPMI-1640 media. All cell lines were routinely tested for mycoplasma contamination.

### Isolation of exosomes

HEK293T cells were cultured in DMEM media supplemented with 10% exosome depleted FBS. After 48 h, conditioned media was collected and used for isolation of exosomes by differential ultracentrifugation. Conditioned media was subjected to an initial centrifugation at 2000×*g* for 30 min. Next, the supernatant was passed through a 0.2 µm filter followed by ultracentrifugation at 100,000×*g* for 2 h in a Beckman Coulter ultracentrifuge with 45T rotor. The obtained pellet of vesicles was resuspended in sterile PBS and stored in aliquots at − 80 °C.

### Exosome quantitation and size determination by nanoparticle tracking analyses

To confirm the integrity of exosome preparations, Nanoparticle Tracking Analysis (NTA) was performed to evaluate particle size and concentration as described earlier^23,45^. NTA analyses were performed using a NanoSight LM10 instrument (Malvern Instruments) equipped with a 405 nm laser as per manufacturer’s instructions. Exosome samples were diluted 1:1000 in PBS and the sample chamber was filled with PBS diluted sample. Each sample was analyzed with NTA 3.0 software, and each analysis consisted of five 30-s .avi (audio video interleaved) file recordings.

### Loading of exosomes with tazemetostat and enzalutamide

HEK293T exosomes** (**10^11^ particles/ml) were mixed with tazemetostat (1µM) (HY-130803, MedChemExpress) and enzalutamide (10 µM) (MDV-3100, Biovision) in 1ml of PBS followed by sonication. Sonication settings used were 20% amplitude, 500V voltage and 2kHz frequency. 6 cycles of sonication were used with 30 s ‘pulse on’ and 2 min of ‘pulse off’. After sonication, exosome samples were incubated at 37 °C for 1 h for recovery. Subsequently, the exosomes were purified using the Total exosome isolation kit (Invitrogen, cat no. 4478359) according to the manufacturer's instructions. Finally, the purified exosomes were visualized and quantified using a Nanosight LM10 instrument (Malvern Instruments).

### Labeling of drug loaded exosomes with CEACAM5 antibody and biotin FITC

To engineer exosomes with CEACAM5 antibody on the surface, we employed a modular EV membrane anchoring platform consisting of streptavidin (STVDN) conjugated with 1,2-bis(dimethylphosphino)ethane: polyethylene glycol 5k (DMPE-PEG) (NANOCS, Inc.), abbreviated as DPS. This platform utilizes DMPE-PEG to embed into the EV membrane, providing a stable anchor for the attachment of fluorescent moieties or antibodies^38^. In two separate reactions, DPS was conjugated with biotin-PEG-FITC (NANOCS, Inc.) and with biotin-CEACAM5 antibody/biotin-IgG. First, the DPS anchor was incubated with the biotinylated molecule in a 1:5 ratio, e.g., 10μg DPS plus 50μg biotinylated-FITC (NANOCS, cat# PG2-BNFC-5k) or biotinylated-CEACAM5 antibody (LSBio, LS-C425805) or biotinylated IgG (LSBio, LS-C60618) for 10 min at 25 °C. Next, the complex was mixed with tazemetostat + enzalutamide labelled HEK 293T exosomes (10^10^–10^11^ particles in 500 μl) and incubated for 10 min at 37 °C. The resulting suspension was concentrated by column concentrator (Pierce, catalog no. 88512). The flow-through (bottom of column, containing unincorporated complexes and dyes) was discarded and the retentate (top of column, containing the engineered EVs) was collected. Engineered EVs were measured and visualized on Nanosight LM10 instrument (Malvern Instruments).

### Immunogold labeling of CEACM5 Ab decorated exosomes

Exosomes were fixed in 4% paraformaldehyde in 0.1 M cacodylate buffer pH 7.4 overnight and 20 µl of suspended exosome preparation was applied to a carbon-formvar coated 200 mesh nickel grid (Electron Microscopy Sciences) and allowed to stand 30 min. Excess sample was wicked off onto Whatman filter paper. Grids were then floated exosome side down on 20 µl drops of 1 M ammonium chloride for 30 min to quench aldehyde groups from the fixation step, followed by flotation on drops of blocking buffer (Electron Microscopy Sciences) for 1 h. After three rinses in PBS, grids were incubated for 1 h on drops of 1.4 nm anti-rabbit nanogold (Nanoprobes, Inc.) diluted 1:1000 in blocking buffer, then washed in PBS three times, for 5 min each wash, followed by three washes in deionized H_2_O. Nanogold was enhanced for 8 min in HQ Silver, (Nanoprobes, Inc.) and rinsed in ice cold deionized H_2_O to terminate enhancement. Grids were then negatively stained in 2% aqueous uranyl acetate and allowed to air dry before being imaged in a JEM 1400Flash transmission electron microscope (JEOL USA Inc.) at 120 kV with a Gatan One View Digital Camera (Gatan Inc.).

### Exosome treatment of cell lines and viability assays

Cell lines were cultured in exosome depleted media in 24-well culture plates. Cells were treated with control exosomes/test exosomes (10^8^ particles/ml media) for 4 days. Following this treatment, cells were plated in 96-well plates and assayed for cell viability using the CellTiter 96 AQueousOne Solution Cell Proliferation Assay Kit (Promega), according to the manufacturer's protocol. For uptake/binding assay, engineered IgG/CEACAM5 Ab labelled HEK 293T exosomes with biotin-FITC tag were incubated with NCI-H660 cells for 30 min. Following binding, NCI-H660 cells were harvested, washed and resuspended in PBS. Cells were visualized and photographed on Keyence microscope.

### In vivo xenograft studies

Animal studies were approved by Augusta University Institutional Animal Care and Use Committee (IACUC) and were performed in accordance with institutional guidelines under an approved protocol (Protocol no. 2019-1013). The studies were done in agreement with ARRIVE (Animal Research: Reporting of In Vivo Experiments) guidelines. Animals were euthanized by isoflurane inhalation followed by cervical dislocation in accordance with IACUC guidelines. To determine the potential anti-cancer effects of engineered CEACAM5-targeted exosomes in NEPC, we employed NE PDX model LuCaP145.1^46^. This PDX model was procured from Dr. Eva Corey at University of Washington. 6 weeks old FOX-Chase SCID male mice (n = 8) were implanted with LuCaP145.1. Once subcutaneous tumors were established, animals were randomized into two groups: Control and Test (n = 4/group). Test mice were administered 10^9^ particles of engineered exosomes (CEACAM5 Ab labelled + tazemetostat + enzalutamide loaded HEK293T exosomes) via tail vein twice a week for 10 days. Controls included LuCaP145.1 xenografts treated with 10^9^ particles of control exosomes (IgG labelled HEK293T exosomes). Average tumor sizes at exosome treatment initiation were 108 mm^3^ in control group and 209 mm^3^ in test group. Tumor volumes were measured regularly and percent tumor growths were calculated by the formula: Volume = 0.5 (length × width^2^). At the end of treatment, mice were sacrificed and tumors were harvested. The potential for observer bias was reduced as the tumor measurements were performed by a person blinded to the treatment group. The humane endpoints of euthanasia included in our animal protocol were used in this study. These endpoints included: Animals showing signs of distress or infection or sepsis; tumors exceeding maximum size of 20 mm in any one dimension; ulcerated tumors; 15% reduction of body weight or < 2 body condition score (bcs) which was monitored on a weekly basis.

### In vivo biodistribution studies

Biodistribution studies with engineered exosomes were performed following the protocol as described in^47^. IgG- or CEACAM5 antibody tagged exosomes were labeled with Iodine-131 radioisotope (I-131) using iodination beads followed by in vivo SPECT studies. Radioisotope-labeled exosomes (3–5 × 10^9^ particles) were administered intravenously and SPECT-CT images were obtained at 3 h. After the intravenous injection of 300 ± 50 µCi of I-131-labeled EVs in 100–200 µl into the tail vein of the mice, whole body SPECT images, were acquired using a dedicated 4-headed NanoScan, high-sensitivity microSPECT/CT 4R (Mediso) fitted with high-resolution multi-pinhole (total 100) collimators. The microSPECT has a wide range of energy capabilities from 20 to 600 keV, with a spatial resolution of 275 µm. The images were obtained using 60 projection images with 30–60 s/projection, with a medium field of view. Attenuation was corrected using concurrent CT images, and then the images were reconstructed with low iteration and low filtered back-projection. During the whole procedure, the animals were anesthetized. Throughout the scanning their body temperature was maintained at 37 °C and breathing was monitored. As a control, we also injected free I-131 in control mice to see the uptake of free I-131.

### Western blotting

Protein extracts were prepared by lysing the cells in RIPA buffer (Invitrogen), which consisted of 50 mmol/l Tris (pH 8.0), 150 mmol/l NaCl, 0.5% deoxycholate, 0.1% SDS, and 1.0% NP-40. 1X Protease inhibitor cocktail (Thermo Scientific) was included during cell lysis. Proteins were quantified by BCA Protein Estimation Kit (Thermo Scientific). 100 µg of each protein extract was loaded on 4–20% mini-PROTEAN TGX gels (Biorad). Western blotting was performed following established protocols. The antibodies and dilutions employed for Western blotting are provided in Table S1.

### Immunohistochemical analyses and H&E staining

Harvested tumors were embedded, sectioned and stained with H&E as per protocol described in^48^. Immunostaining was done on formalin-fixed, paraffin-embedded LuCaP145.1 xenograft tissues treated with control/test exosomes. Slides were deparrafinized, antigen retrieval was carried out by microwaving the slides in 10 mmol/l sodium citrate buffer followed by overnight incubation with primary antibody. Antibodies included ENO2 antibody (Catalog no. 65162S) (Cell Signaling), SYP (Catalog no. MA5-14532), CHGA (Catalog no. MA5-13096), Ki67 (Catalog no. MA5-14520) and CEACAM5 (Catalog no. LS-C345349) (Table S1). Following washes with PBS, anti-rabbit secondary antibody was added, and slides were incubated for 1 h at room temperature. Slides were washed with PBS and developed with DAB staining kit (Immpact, Vector Laboratories, Newark, CA) following manufacturer’s instructions. The slides were counterstained with hematoxylin. Histoscore (H-score) was calculated for each stained sample for IHC assessment. Based on staining intensity, stained tissue sections were assigned a score of 0 (no staining), 1 (weak staining), 2 (moderate staining), 3 (strong staining). The percentage of positively stained cells for each intensity level were calculated. Then H-scores were calculated using the formula: H-Score = (percentage of cells with intensity 1 * Intensity 1) + (percentage of Cells with intensity 2 * intensity 2) + (percentage of cells with intensity 3 * intensity 3). Following this, average H-scores for controls vs test samples were calculated along with standard deviation. Statistical analyses was conducted by Two Way ANOVA followed by Bonferroni multiple comparison post-hoc test.

### Statistics

All quantified data represents an average of triplicate samples or as indicated. Experiments with cell lines included at least three biological replicates and two to three technical replicates. Data are represented as mean ± S.E.M or as indicated. Statistical significance between groups was assessed by Student's t-test. For in vivo studies, statistical significance between control/test groups was assessed by One-Way ANOVA. Statistical analyses were performed using GraphPad software version 10. Results were considered statistically significant at P ≤ 0.05.

### Supplementary Information

Supplementary Information.

## Data Availability

Data generated or analyzed during this study are included in this article and its supplementary information files.
