# Supplementary material for: A novel exosome based therapeutic intervention against neuroendocrine prostate cancer
Source: Sci Rep. 2024 Feb 2;14:2816. doi: 10.1038/s41598-024-53269-9 (PMC10837194; doi:10.1038/s41598-024-53269-9)

Supplemental Data

<u>Supplemental Results:</u>

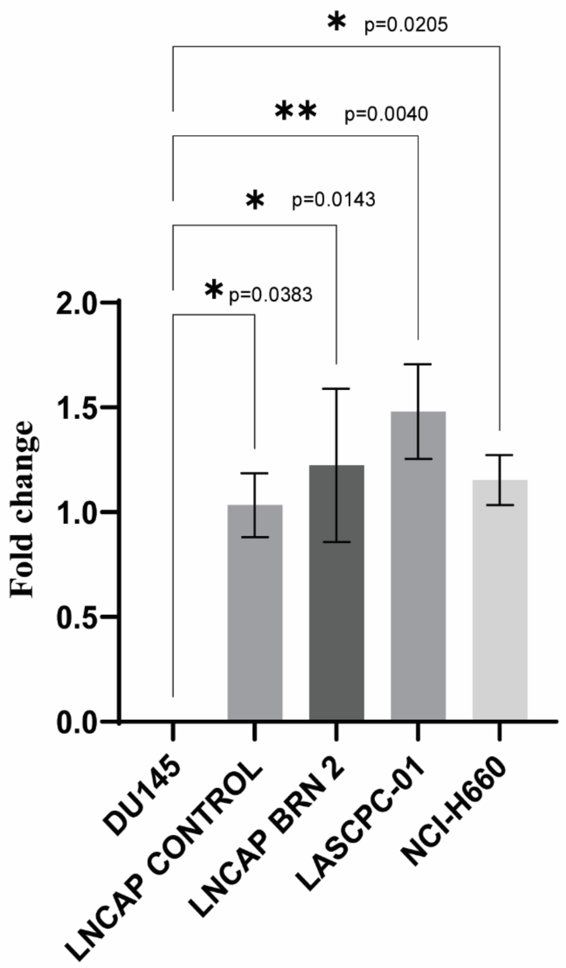

Fig. S1 Quantification of CEACAM5 expression in various prostate cancer cell lines.
Western blot analyses of CEACAM5 expression (Fig. 1E) was used for Image J analyses. Three biological replicates were analyzed and relative quantification was calculated by dividing CEACAM5 band intensities with that of GAPDH.. Statistical analyses was performed by One Way ANOVA and p-values were calculated relative to Du145.

Fig. S2 MTS assay in RWPE-1 cells after treatment with Tazemetostat+enzlautmaide loaded exosomes

HEK293T exosomes were loaded with tazemetostat (1µM) and enzalutamide (10µM) by sonication. Purified control exosomes or drug loaded exosomes (10^8 particles) were used for treating RWPE-1 cells for 4 days. Shown are the relative cellular viabilities in control exosome/drug loaded exosome treated cells as assessed by MTS cellular viability assay. Drug loaded exosomes did not affect viability

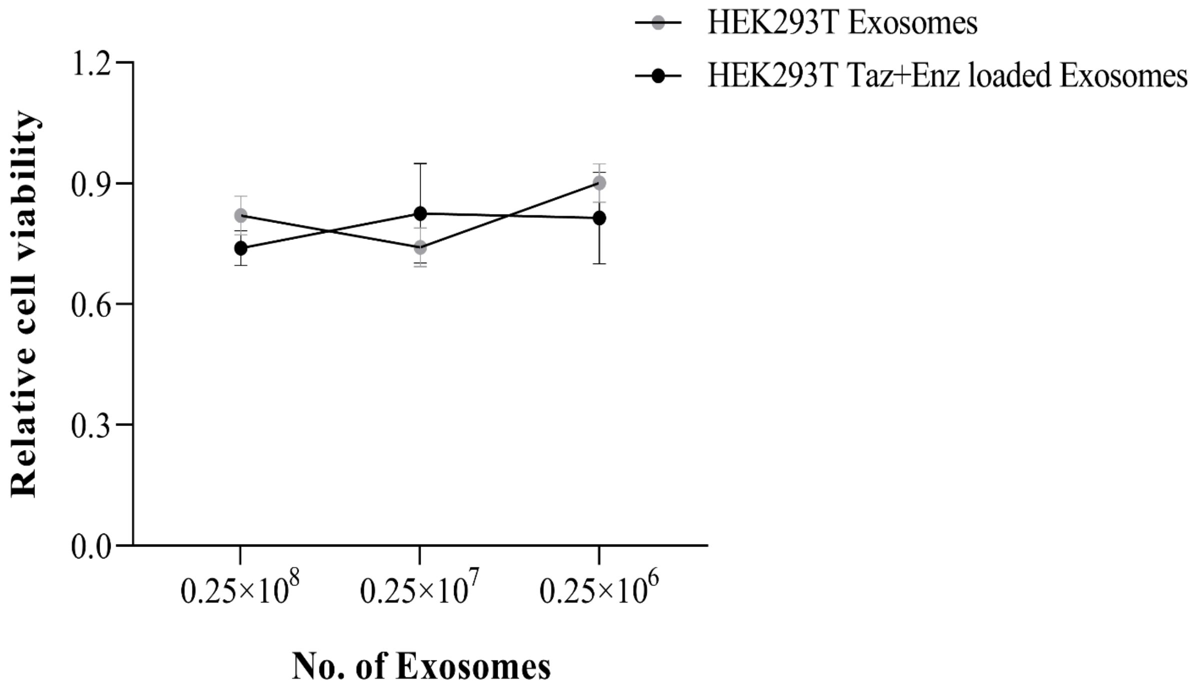

in these cells similar to NEPC cells.

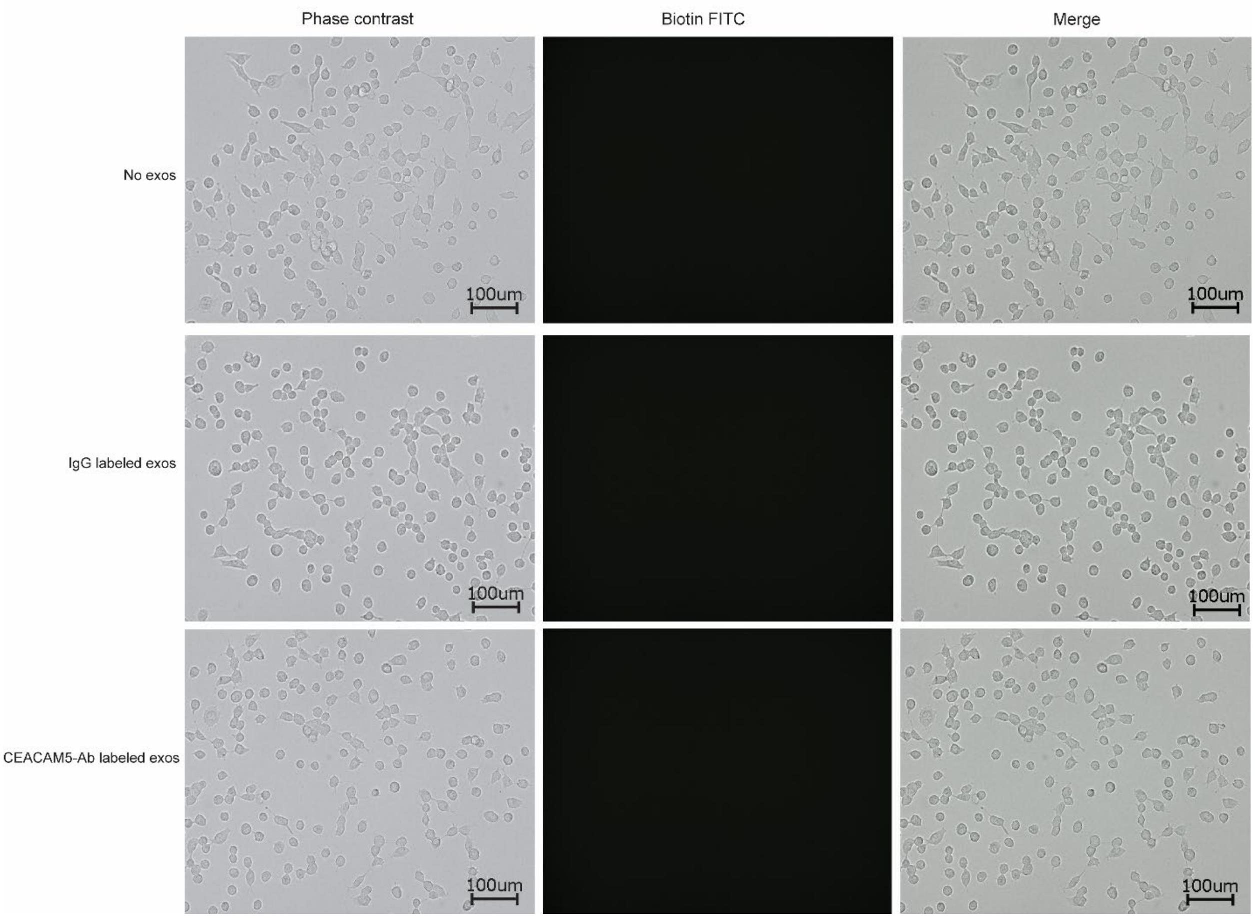

Fig. S3 Binding Affinity Assay of Engineered CEACAM5-targeted exosomes in RWPE1 cell line. RWPE-1 cells were treated with IgG labelled exosomes as seen in the middle panels and CEACAM5-Ab labelled HEK293T exosomes (lower panels). No exosome treated RWPE-1 cells were included as observed in the upper panel. This attests that CEACAM5 targeted exosomes adhere specifically to NEPC cells.

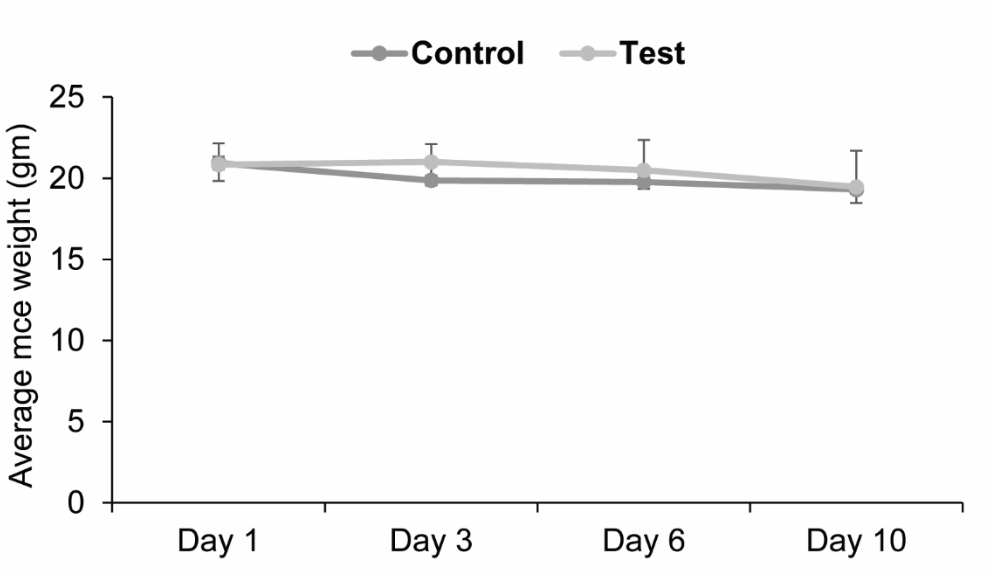

Fig. S4 Mice weight in control and test groups upon exosome administration

LuCaP145.1 tumors were established in FOX-Chase SCID mice. Once established, mice were divided into two groups: Control and Test. Test mice were administered 10^9 particles of engineered exosomes (CEACAM5 Ab labelled + tazemetostat + enzalutamide loaded HEK293T exosomes) via tail vein twice a week for 10 days. Controls included LuCaP145.1 xenografts treated with 10^9 particles of control exosomes (IgG labelled HEK293T exosomes). Mice weight were calculated at each time point and plotted.

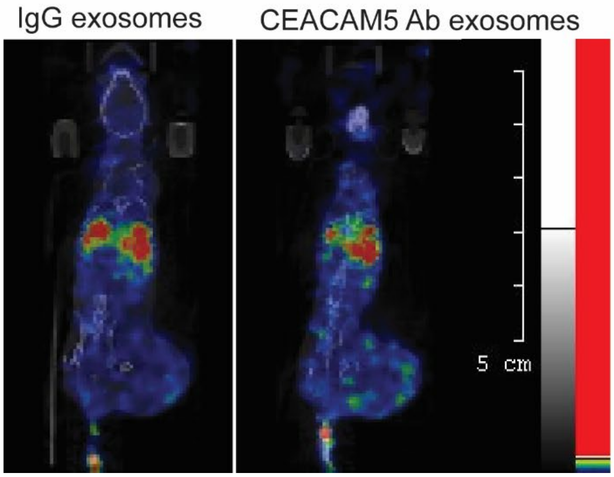

Fig. S5 Biodistribution studies of CEACAM5-targeted exosomes in LuCaP145.1 PDX model

Control IgG exosomes or CEACAM5 antibody loaded exosomes were radiolabeled with Iodine-131. Radiolabeled exosomes were administered via the tail vein into mice bearing LuCaP145.1 tumors followed by in vivo SPECT imaging after 3 hours of exosome injections.

Table S1: List of Antibodies

Primary antibody	Source of purchase	Cat. No.	Dilutions	Application
CD9	Cell Signaling Technology, Inc.	13174S	1:1000	WB
CD63	Life Technologies Corp.	10628D	1:1000	WB
CEACAM5	Lifespan Biosciences Inc.	C425805	1:250	WB, IHC
GAPDH	Cell Signaling Technology, Inc.	2118S	1:1000	WB
BRN2	Cell Signaling Technology, Inc.	12137S	1:1000	WB
ASCL1	Cell Signaling Technology, Inc.	10585S	1:1000	WB
BRN4	MilliporeSigma	HPA31984	1:1000	WB
ENO2	Cell Signaling Technology, Inc.	65162S	1:1000	WB, IHC
SYP	Thermo Fisher Scientific	RM-9111-S	1:200	WB, IHC
CHGA	Invitrogen	MA5-13096	1:200	IHC
Ki67	Invitrogen	MA5-14520	1:1000	IHC
Cleaved PARP	Cell Signaling Technology, Inc.	9541S	1:1000	IHC
Cleaved Caspase-3	Cell Signaling Technology, Inc.	9661S	1:1000	IHC
CD44	Cell Signaling Technology, Inc.	3570S	1:1000	IHC
E-Cadherin	Cell Signaling Technology, Inc.	3195S	1:1000	IHC

WB, Western blot; IHC, Immunohistochemistry

<u>Supplemental Methods:</u>

Power Analyses

For in vivo studies, power analyses was performed with G-power Version 3.1 as below:

t tests - Correlation: Point biserial model

**Analysis:**	Post hoc: Compute achieved power		
**Input:**	Tail(s)	=	One
	Effect size $\lvert\rho\rvert$	=	0.8
	α err prob	=	0.05
	Total sample size	=	8
**Output:**	Noncentrality parameter δ	=	3.7712362
	Critical t	=	1.9431803
	Df	=	6
	Power (1-β err prob)	=	0.9517176

Western blots used in the manuscript

Enolase 2 for IgG exosomes and CEACAM5 antibody labeled exosomes as shown in Fig. 4

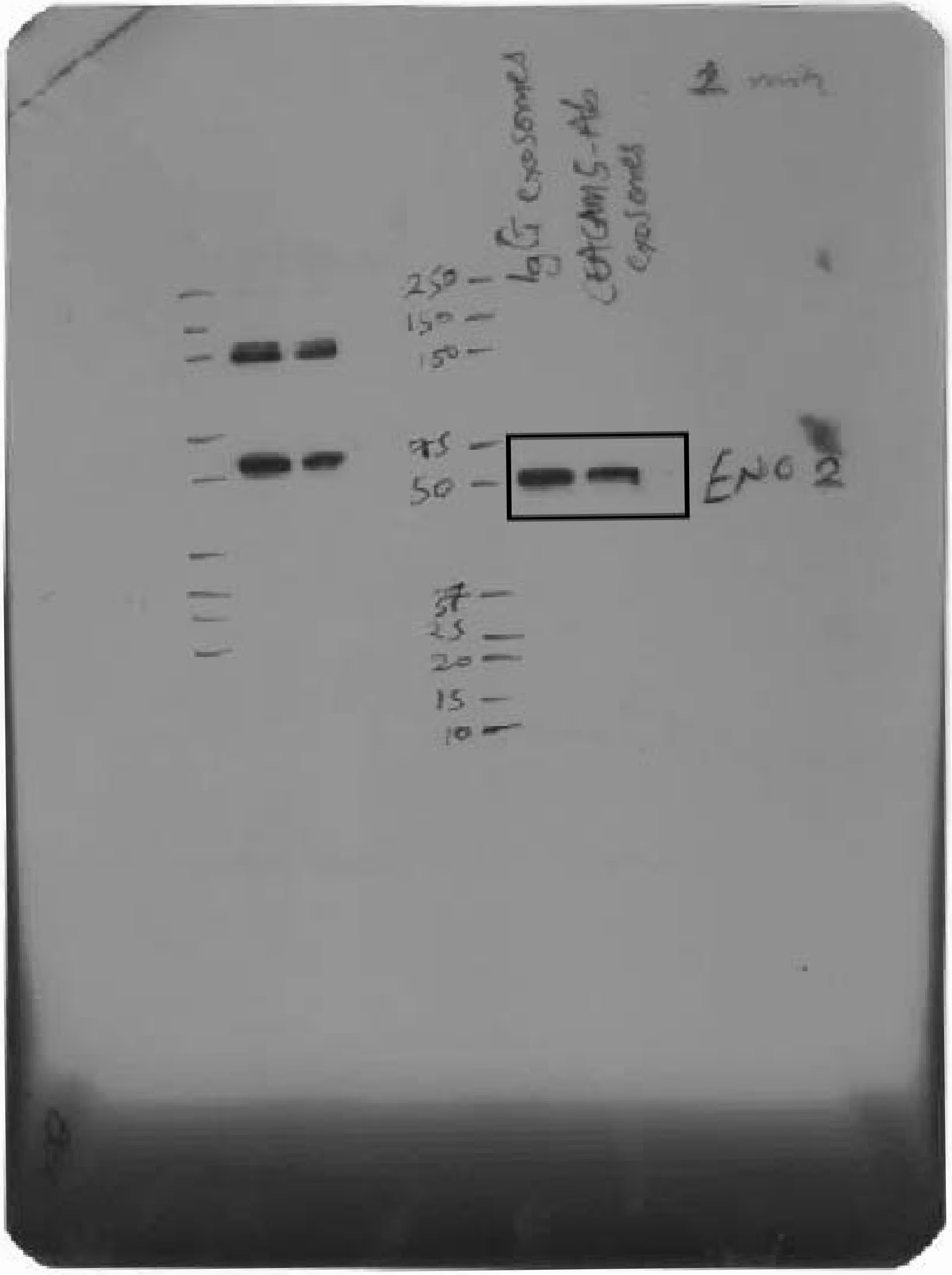

BRN2, ASCL1 and Synaptophysin for IgG exosomes and CEACAM5 antibody labeled exosomes as shown in Fig. 4

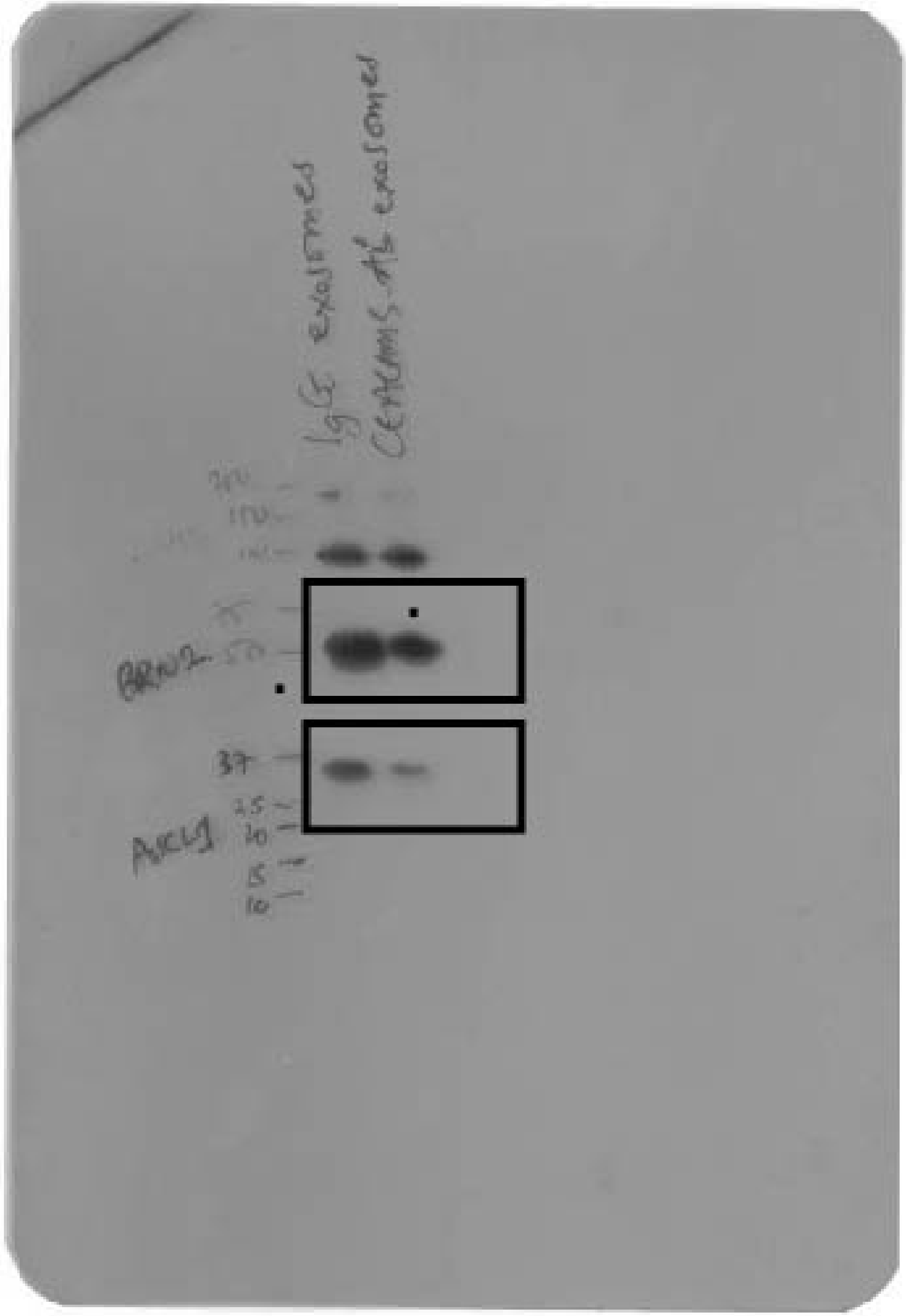

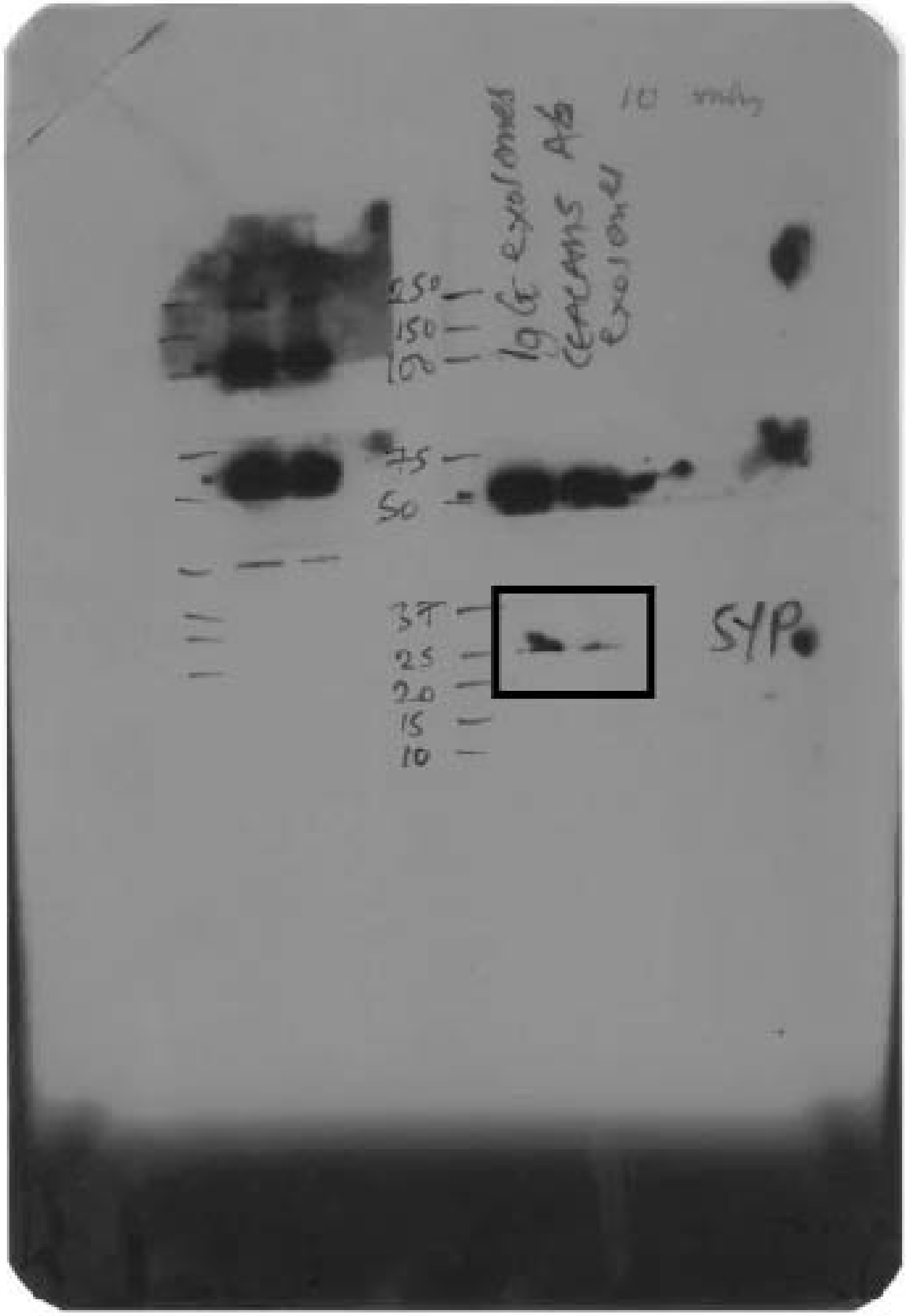

BRN4 for IgG exosomes and CEACAM5 antibody labeled exosomes as shown in Fig. 4

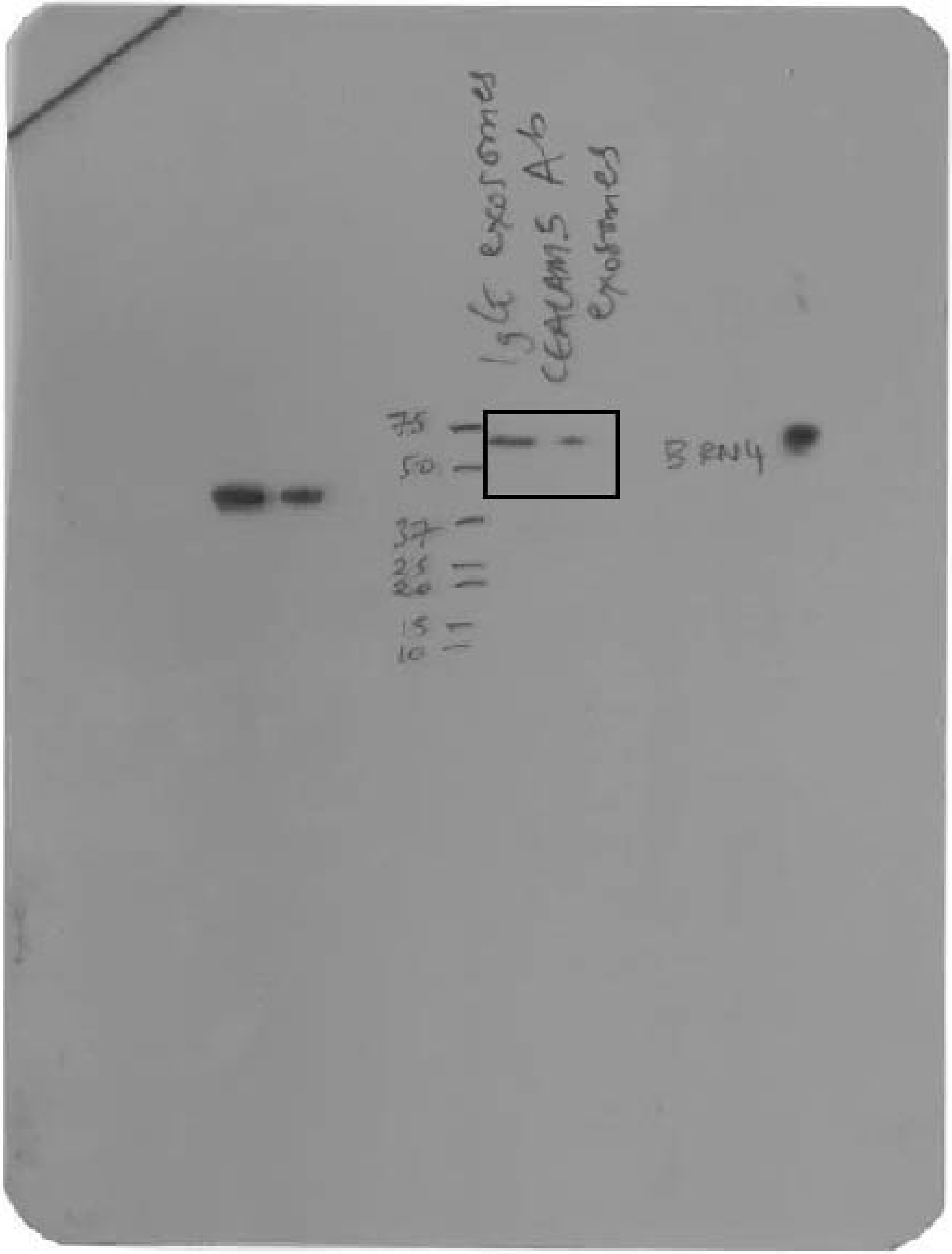

GAPDH for IgG exosomes and CEACAM5- Ab labeled exosomes in ASCL1 and BRN2 blot as shown in Fig. 4

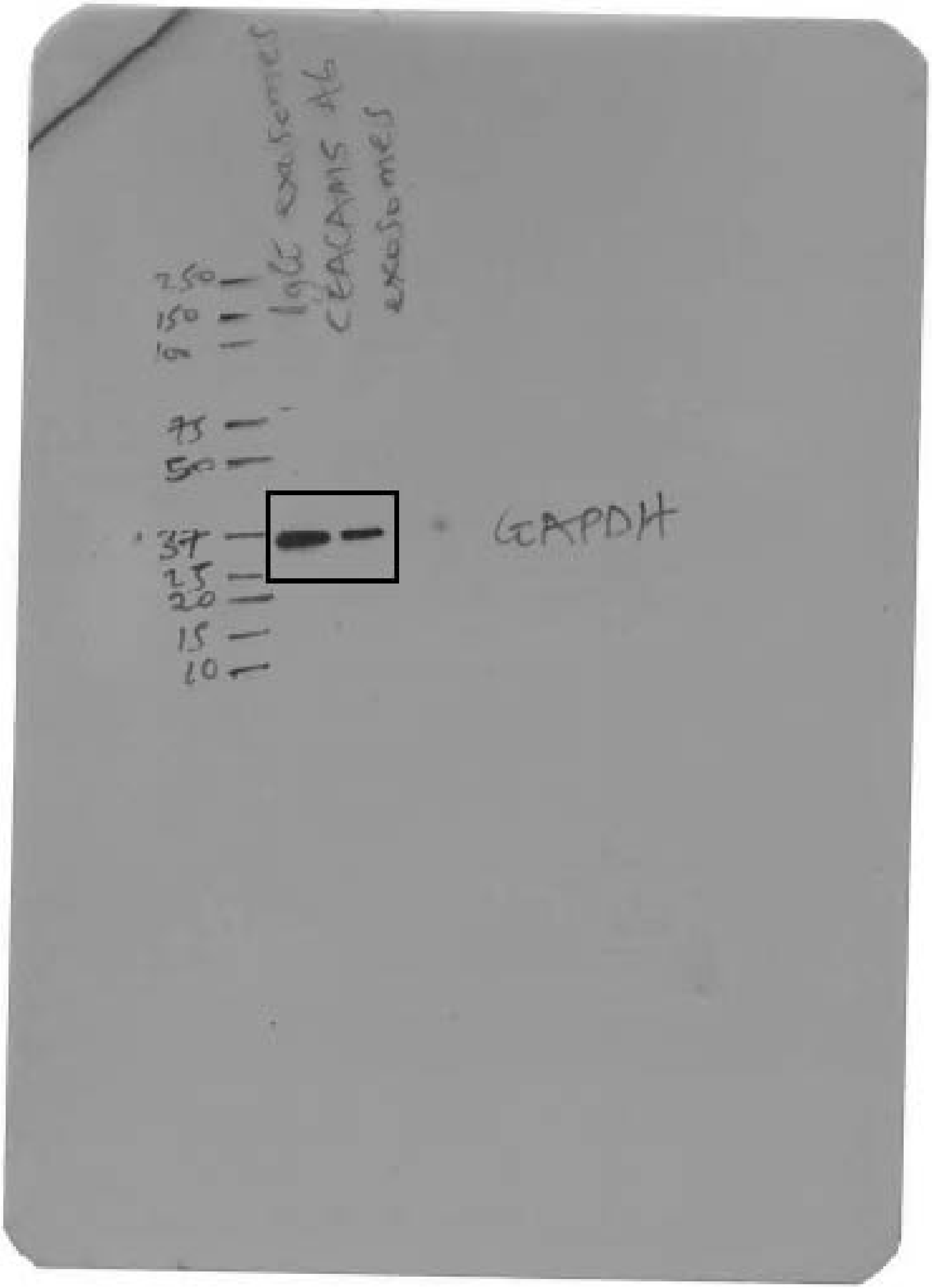

GAPDH for IgG exosomes and CEACAM5-Ab labeled exosomes in BRN4, Enolase 2 and Synaptophysin blot as shwon in Fig. 4

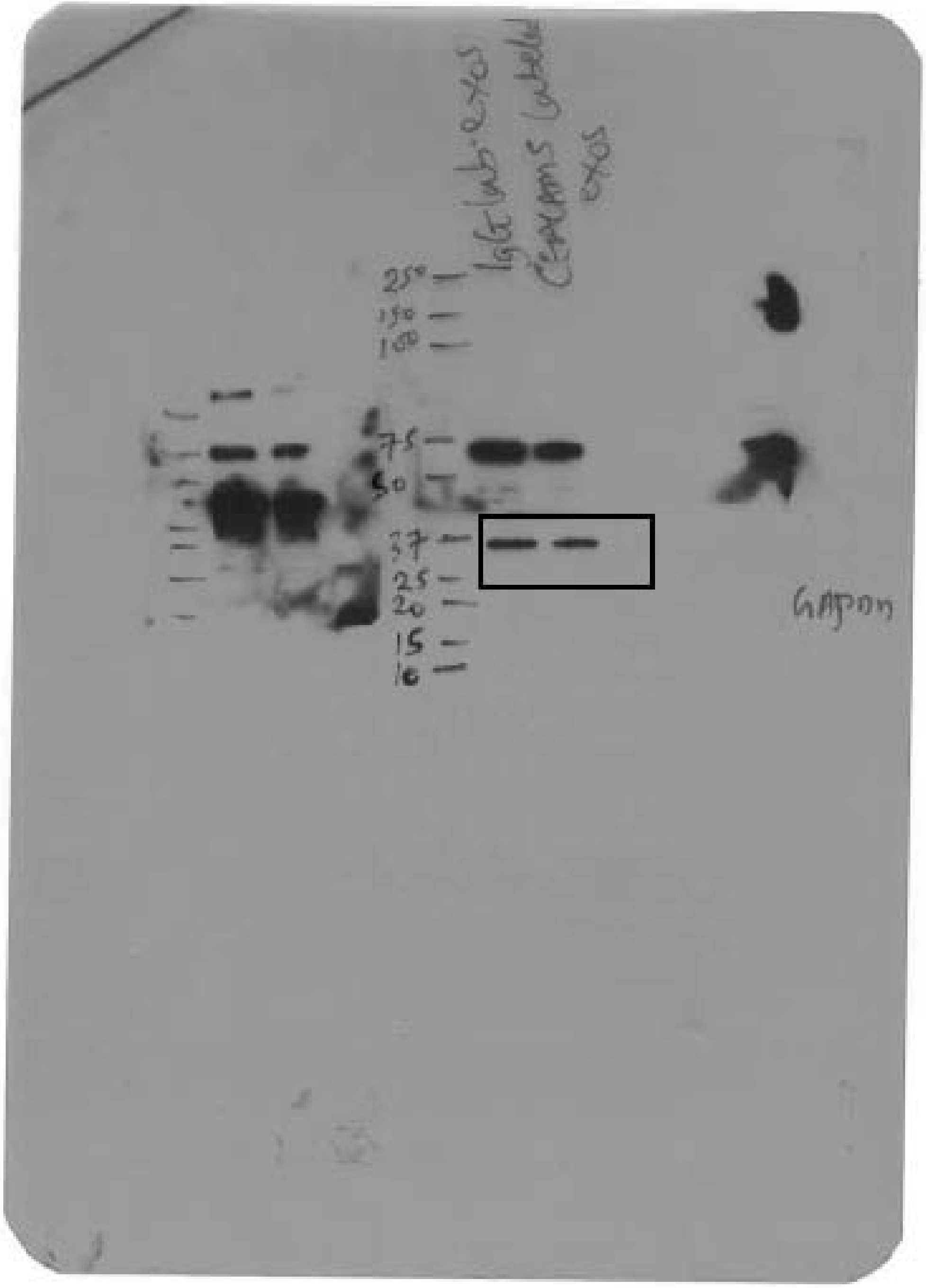

BRN2 and GAPDH for LNCaP control and LNCaP cells over-expressing BRN2 as shown in Fig. 1

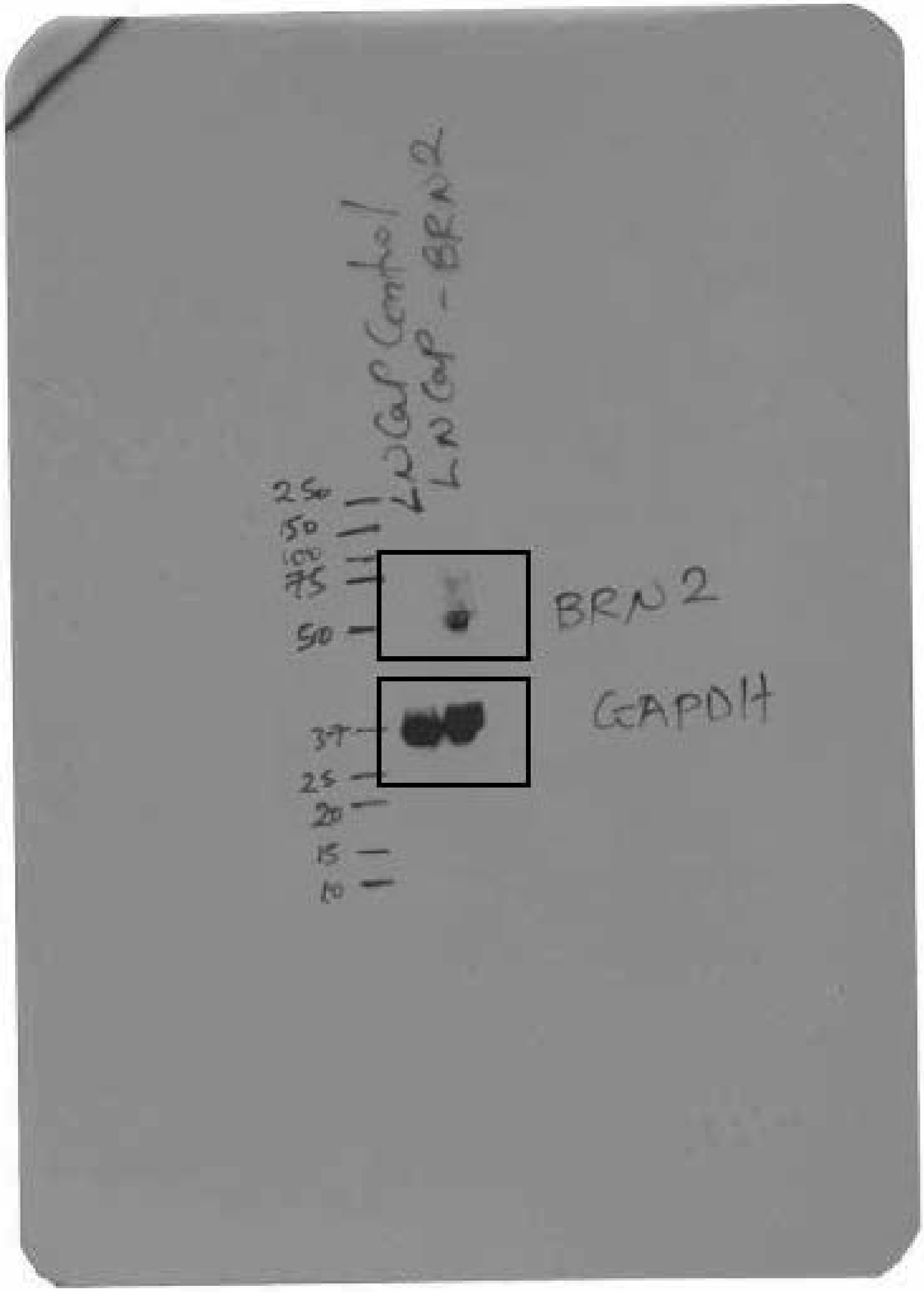

CD63 for HEK293T exosomes and drug-labeled exosomes as shown in Fig. 1

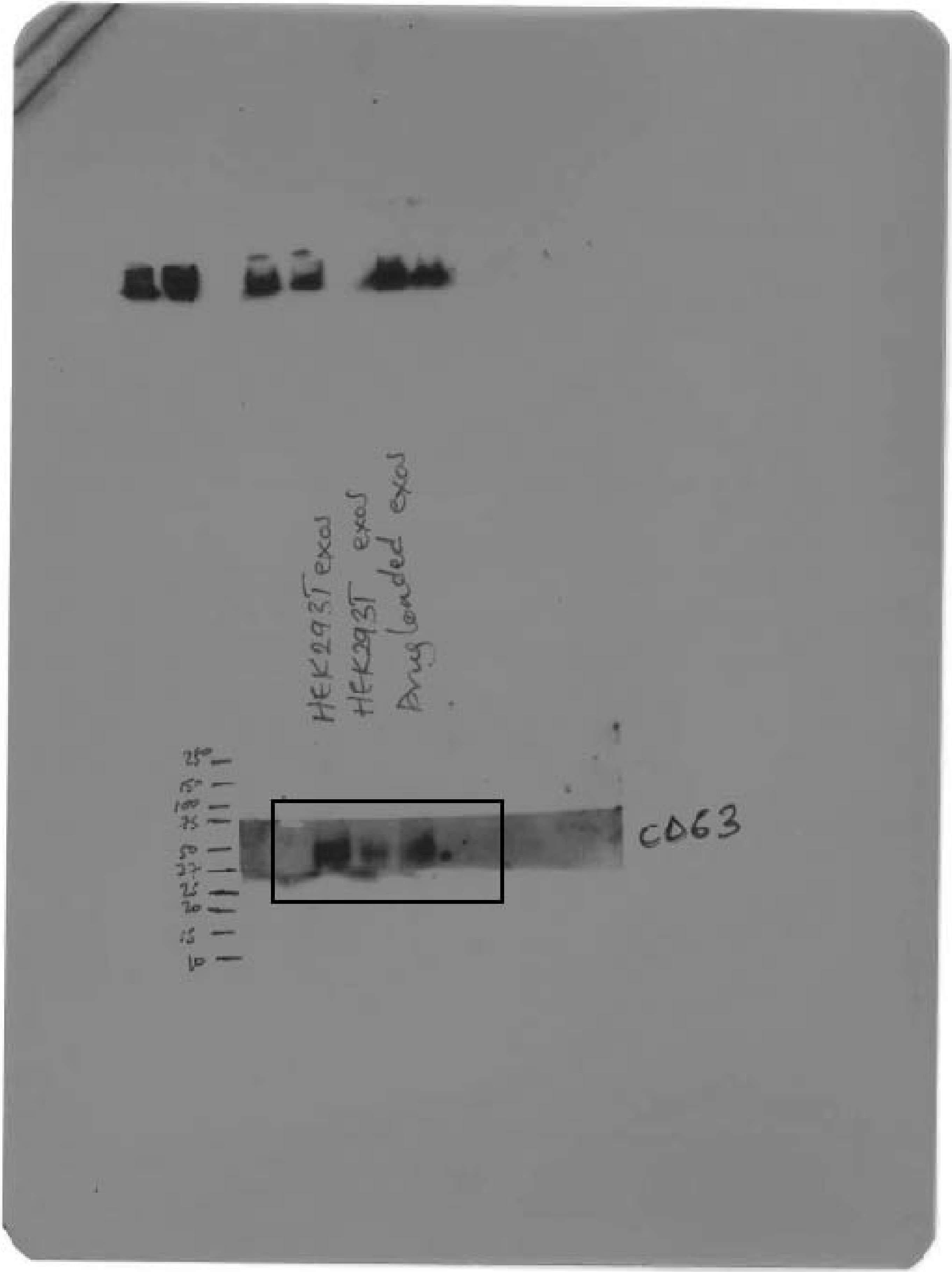

CD9 for HEK293T exosomes and drug-labeled exosomes as shown in Fig. 1

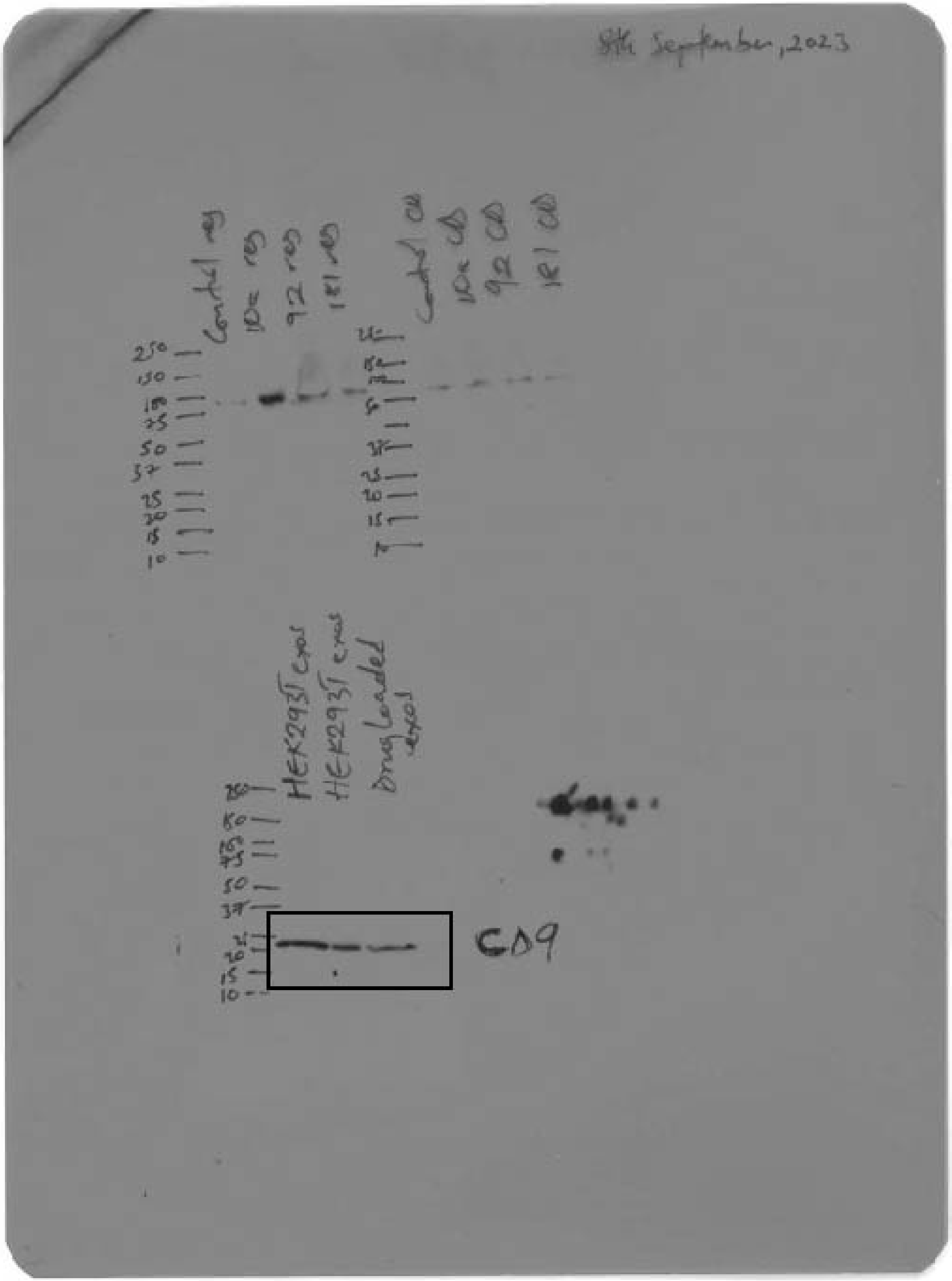

CEACAM5 and GAPDH for DU145, LNCaP control, LNCaP over-expressing BRN2, LASCPC-01, and NCIH660 cells as shown in Fig. 1

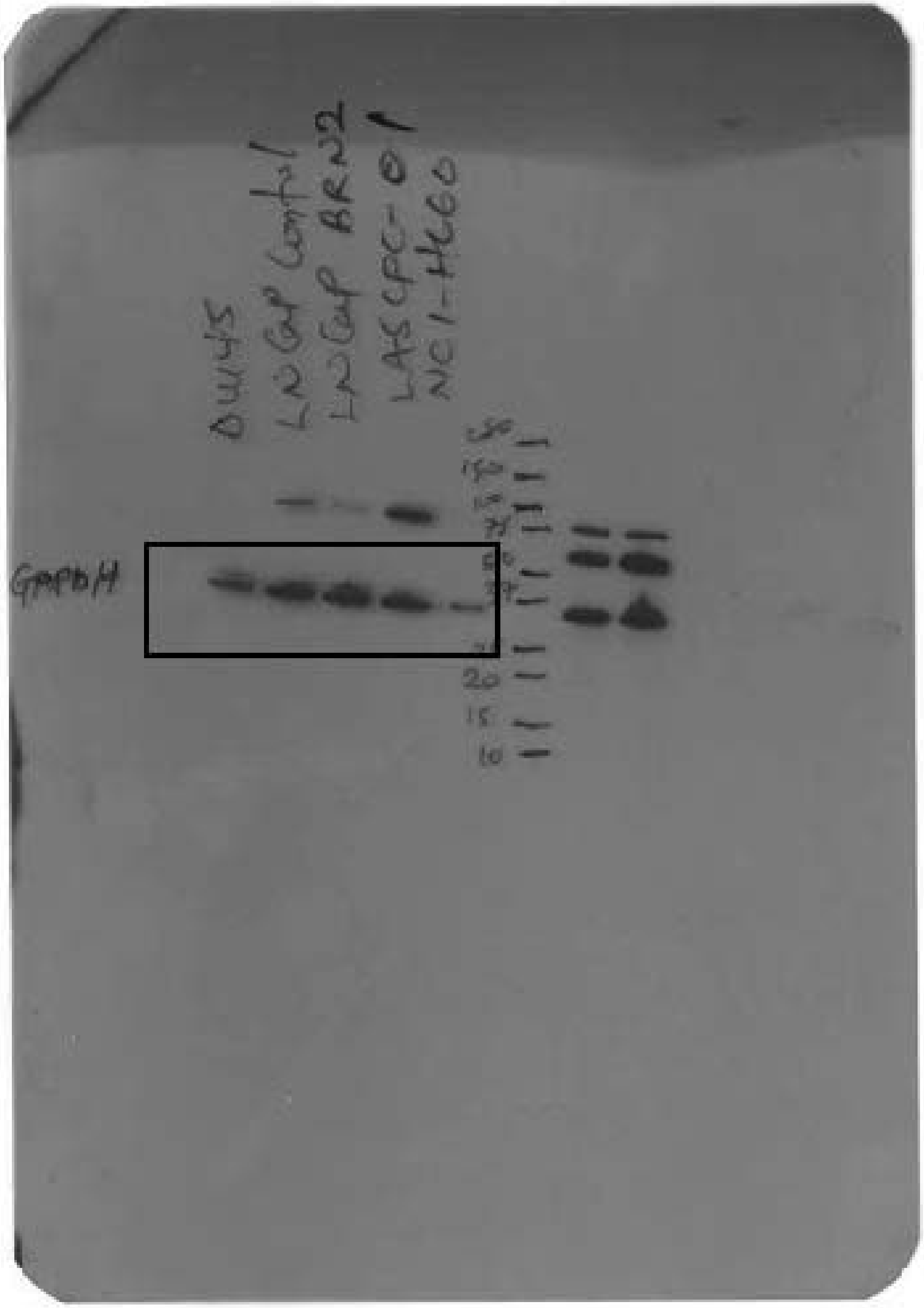

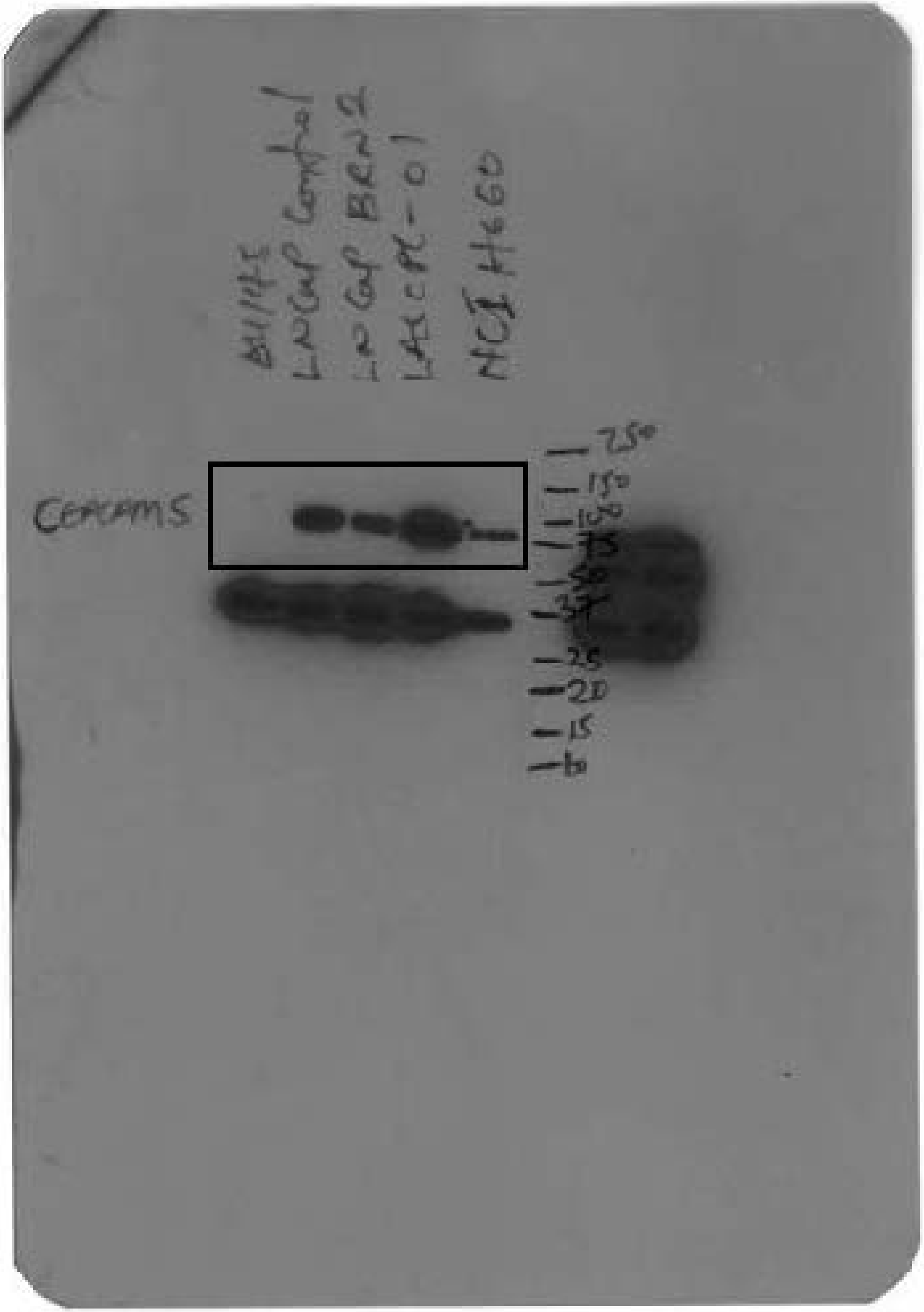

Supplement: Supplementary file 1 — Supplementary Information. [file 41598_2024_53269_MOESM1_ESM.pdf]
